# Norm Entrepreneurship in Digital Trade: The Singapore-led Wave of Digital Trade Agreements

**DOI:** 10.1017/S1474745624000089

**Published:** 2024-05

**Authors:** Emily Jones, Beatriz Kira, Rutendo Tavengerwei

**Affiliations:** 1 Blavatnik School of Government, University of Oxford, Oxford, United Kingdom; 2 School of Law, University of Sussex, Brighton, United Kingdom; 3 Faculty of Law, University of Oxford, Oxford, United Kingdom

**Keywords:** digital trade, Singapore, norm-entrepreneurship

## Abstract

Rulemaking in digital trade is proceeding apace. Many preferential trade agreements contain dedicated e-commerce or digital trade chapters and some states have entered into stand-alone digital economy agreements. This article seeks to establish whether, and to what extent, normative change is occurring in digital trade agreements, the nature of any changes, and identify which states are acting as norm entrepreneurs. We employ a new method of legal coding, systematically comparing the nature and prescriptiveness of digital provisions in 12 trade agreements concluded between 2019 and 2023. We find evidence of substantial policy innovation, and identify Singapore as the key norm entrepreneur. A new wave of ‘Singapore-led’ agreements substantially expands the scope of digital trade, to cover areas such as digital identities, e-invoicing and e-payments, the governance of AI, and regulation of new digital technologies. Commitments are typically couched as soft rather than hard law, reflecting the nascent stages of rulemaking. Norm entrepreneurship on the part of Singapore and its allies reflects a desire to position themselves as ‘digital hubs’ in the global economy, spur rulemaking in areas where innovation is ahead of regulation, and promote digital interconnectivity at time of regulatory divergence and geopolitical rivalry.

## 1. Introduction

Digitalization is transforming international trade, changing the nature of cross-border economic interactions.^1^ An increasing proportion of goods and services are ordered and delivered virtually, data have become a major cross-border economic flow, and digital technologies are embedded throughout supply chains. Meanwhile, the internet has enabled the emergence of platform companies of unprecedented size and global reach, and allowed a growing number of small businesses, individual workers, and consumers to engage in cross-border trade.

These shifts have created new cross-border opportunities and challenges, spurring governments to negotiate new trade rules. The range of issues is broad, ranging from the governance of cross-border data flows and the digitalization of customs documents to the enhanced protections for source code. Discussions started at the World Trade Organization (WTO) in 1998 but made little progress,^2^ so governments turned to preferential trade agreements, initially including a handful of provisions and then expanding the scope to include dedicated chapters.^3^ By the end of 2022, there were 167 preferential agreements containing provisions on digital trade, with 109 containing dedicated chapters.^4^

Among the three major blocs in the world economy, the US has embraced the move to negotiate digital trade rules, while the EU and China have been much more hesitant.^5^ From the early 2000s, the US used trade agreements to pursue its ‘Digital Agenda’, and was central in digital trade rule-making. More than half of all new provisions on digital trade, introduced between 2000 and 2019, were in trade agreements where the US was a signatory.^6^ Digital trade was a major priority of the Obama administration, reflected in the extensive digital trade provisions in the Trans-Pacific Partnership agreement (TPP), which was signed in 2016. (This later became the Comprehensive and Progressive Agreement for Trans-Pacific Partnership (CPTPP) in 2018, following the Trump administration decision to withdraw).^7^ The TPP was followed by the US–Mexico–Canada Agreement (USMCA), concluded in 2018, and the Japan–US Digital Trade Agreement in 2019. The US focused on the liberalization of data flows, and the removal of other government measures deemed by large US technology companies to be impediments to their business model, including the so-called ‘forced transfer’ of software and source code. Through these agreements, the US claimed it had set a new ‘gold standard’ for digital trade rules.

With the expiry of the US Trade Promotion Authority in 2021, and an initial turn away from trade agreements under the Biden administration, the US has been less of a driving force in shaping digital trade rules. Yet other governments have continued to negotiate extensive digital commitments, including the preferential trade agreements negotiated by the UK after Brexit. In 2020, Chile, New Zealand, and Singapore negotiated the world's first stand-alone digital economy agreement, with the explicit aim of shaping global norms.

This article seeks to establish whether, and to what extent, recent digital trade agreements replicate the earlier US approach or depart from it. Moreover, where agreements differ, is this part of a wider pattern of change? Alternatively put, to what extent has normative change been happening in digital trade since the US stepped back, and which states are acting as norm entrepreneurs? Following Finnemore and Sikkink, we identify norm entrepreneurs as states that attempt to influence a critical mass of other states to embrace new norms (standards of appropriate behaviour).^8^ Normative change occurs when these norms become more widely accepted and internalized among states.

To establish whether, and to what extent, norm entrepreneurship is occurring in digital trade, we systematically compare provisions in 12 agreements concluded between 2018 and 2023 that we identify as containing extensive digital provisions. These include three US-led agreements, concluded over 2018 and 2019, and nine other agreements, concluded since then. As explained in detail below, we organize the agreements chronologically and systematically code each provision according to the legal nature of the commitment being made. This enables us to identify the ways in which digital trade commitments have evolved across time, the jurisdictions, and issue areas.

We find evidence of substantial innovation in recent digital trade agreements and identify Singapore as a norm entrepreneur. We show that the most substantial innovation occurred in the Digital Economic Partnership Agreement (DEPA) between Chile, New Zealand, and Singapore (June 2020) and the Digital Economy Agreement (DEA) between Australia and Singapore (Australia–Singapore DEA) (August 2020). Subsequent agreements, including the UK's post-Brexit digital trade agreements incorporated many of these innovations, with some additional adaptations occurring at the margins. While the Singapore-led wave of agreements incorporates core elements of the previous US-led agreements, the recent agreements are more expansive in scope, covering 14 additional issues, including commitments on digital identities, e-invoicing and e-payments, as well as the governance of AI and new digital technologies. There are also drafting novelties in other areas with, for instance, stronger commitments on paperless trade.

While the emphasis of earlier US-led agreements was on promoting the market access of US technology companies and protecting their business models,^9^ we show that the Singapore-led agreements place greater emphasis on promoting regulatory cooperation, technical interoperability between digital systems, and international standard-setting for digital technologies. Reflecting these differing objectives, many digital trade provisions in US-led agreements are hard law commitments, while many of the novel provisions introduced through Singapore-led agreements are couched as soft law.

We find substantial evidence that Singapore intentionally set out to shape global norms, rather than negotiating digital trade agreements simply to enhance its bilateral trade relations. In addition to providing a revised normative framework and set of rules for digital trade, it deployed a variety of strategies to persuade other states to follow, with some success. We attribute norm entrepreneurship on the part of Singapore and its allies to a desire to capitalize on digitalization and position themselves as ‘digital hubs’ in the global economy; spur rule-making in areas of the digital economy where there are gaps and regulatory uncertainties; and promote digital interconnectivity at a time when governments around the world, including the three digital superpowers (the US, EU, and China), were adopting divergent regulatory approaches.^10^

Our work contributes to a growing body of literature that seeks to understand the evolution of digital trade rules. Several scholars have conducted comparative analyses of the US and EU approach to digital trade agreements,^11^ while others, such as Yakovleva and Shaffer, have undertaken comparative analyses of specific issues, contrasting the more liberal approach of the US with the EU's more guarded one.^12^ Some scholars have compared US and EU approaches with those of China,^13^ while others have interrogated the role of the US in shaping the framing and nature of commitments in digital-related trade agreements worldwide.^14^ Other scholars have analysed the interface between digital provisions in bilateral and regional trade agreements in light of commitments under the WTO's GATT and GATS.^15^ Wunsch-Vincent and Hold coded and examined digital provisions in a select number of FTAs concluded between 2000 and 2010, determining the extent of liberalization in these agreements.^16^ Burri and Polanco built on this work by creating a broader dataset of all relevant FTAs concluded since 2000.^17^ Their analysis employs a detailed coding system to map the evolution of digital provisions in trade agreements, giving particular attention to the level of commitment by analysing the strength of the legal language.^18^ The conclusion of DEPA, the world's first stand-alone digital economy agreement in 2020, has spurred discussion on the extent and nature of evolution in digital trade law.^19^

We contribute to this literature by revealing the role played by Singapore in digital trade innovation, helping disrupt the conventional narrative which assumes that the US, EU, and China are the normative drivers of digital policymaking. We provide a systematic and detailed analysis of how the new wave of ‘Singapore-led’ agreements compare to other recent digital trade agreements, showing how the scope of commitments has expanded, and the precise ways in which the legal drafting of commitments compares with the earlier US-led wave. We also contribute to coding methodologies, providing an example of how to systematically incorporate analysis of public policy flexibilities into coding decisions, thereby enhancing our collective understanding of the nature of legal commitments that countries are making in trade agreements.

The article proceeds as follows. In Section 2, we explain our methodology. In Section 3 we present our overall findings, showing that substantial innovation is taking place and propose that a new and distinct ‘Singapore-led’ wave of digital trade agreements is emerging. Drawing on scholarship on norm entrepreneurship, we argue that Singapore is acting as a norm entrepreneur in digital trade, substantiating our argument with a range of primary evidence. In Section 4 we analyse the nature of the ‘Singapore-led’ wave of trade agreements in detail, showing how they compare with the earlier US-wave in coverage and in legal drafting. In the conclusion, we reflect on the progress and prospects of Singapore's norm entrepreneurship in digital trade.

## 2. Research Methods

To understand how digital trade rulemaking has evolved over time, we systematically compare the digital trade provisions in 12 agreements concluded between March 2018 and January 2023. This includes the three major digital agreements that were led by the US (CPTPP,^20^ USMCA,^21^ and the Japan–US Digital Trade Agreement^22^) and nine agreements with substantive digital trade provisions that have been negotiated since then: Digital Economic Partnership Agreement (DEPA) between Chile, New Zealand, and Singapore (June 2020);^23^ Australia–Singapore Digital Economy Agreement (August 2020);^24^ Japan–UK Free Trade Agreement (October 2020);^25^ Regional Comprehensive Economic Partnership Agreement (RCEP) (November 2020);^26^ EU–UK Trade and Cooperation Agreement (December 2020);^27^ Australia–UK Free Trade Agreement (December 2021);^28^ Singapore–UK Digital Economy Agreement (February 2022);^29^ New Zealand–UK Free Trade Agreement (February 2022);^30^ and Korea–Singapore Digital Economy Agreement (January 2023).^31^ While other trade agreements have been concluded during this period, we excluded those without substantial digital chapters from our analysis, as our aim is to understand the scope and nature of the frontier of rulemaking in digital trade rather than to provide an exhaustive analysis of all digital provisions in recent trade agreements.

We extracted provisions on digital trade from these 12 agreements, identifying 34 different issues which we divided into six thematic areas.^32^ The database includes provisions from digital trade chapters as well as provisions in other chapters that specifically relate to digital trade (for instance, commitments on cross-border flows of financial data are often found in the financial services chapter, while commitments on the liability of internet service providers are typically found in intellectual property chapters). Where a digital agreement is being negotiated to update and amend an underlying FTA, we examine the original treaty to ensure we included any provisions that are incorporated (or excluded) by reference.^33^

We code the digital provisions in each trade agreement using a two-part coding system. Our approach draws on recent debates about the nature of legalization in international relations, and the difference between ‘hard’ and ‘soft’ legal obligations. Abbott and Snidal use the term ‘hard law’ to refer to legally binding obligations that are precise (or can be made precise through adjudication or the issuance of detailed regulations) and that delegate authority for interpreting and implementing the law.^34^ ‘Soft law’ is a residual category, referring to legal arrangements that are weakened along one or more dimensions of obligation, precision, and delegation. A treaty is soft in terms of obligation if it is non-binding. It is also soft in terms of precision if it is formally binding but couched in vague language that leaves almost complete discretion to the parties as to its implementation. In contrast, an agreement that fails to delegate any authority to a third party to monitor its implementation or to interpret and enforce it, is soft in terms of delegation.^35^ While trade law is closest to the ideal type of ‘hard law’, it can also be ‘soft’ in important ways. This is particularly true for recent digital trade agreements where technological change is occurring so quickly that commitments may not be ‘treaty-ready’.

The first part of our coding system builds from our discussion on legalization and we assign a numerical value between one and four to capture varying degrees of obligation and precision in individual treaty commitments. In assigning values, we are not making a judgement as to the economic or societal value of the commitment (e.g. whether it is more or less liberalizing, or more or less protective of consumers), but merely assessing its legal nature. Where a commitment is assigned the value of ‘zero’, this means that agreement does not contain a provision on a given issue. The value of ‘one’ is assigned where the agreement contains a provision couched in purely hortatory language such that, even if the treaty as a whole is binding, the individual commitment is non-binding. For example, under ‘regulating cross-border logistics’ in Table 1, DEPA is assigned a ‘one’ because the drafting language is purely hortatory, such as ‘recognize the importance of’ and ‘shall endeavour to’, reflecting the non-binding nature of the commitment.

**Table 1.** Coding of digital provisions in trade agreements concluded between March 2018 and January 2023 (date of signature)

	CPTPP 03/2018	USMCA 11/2018	JPN-US 10/2019	DEPA 06/2020	AUS-SG DEA 08/2020	JPN-UK 10/2020	RCEP 11/2020	EU-UK 12/2020	AUS-UK 12/2021	SG-UK DEA 02/2022	NZ-UK 02/2022	KOR-SG 01/2023
Issue 1: Trade facilitation												
Moratorium on customs duties on e-transmissions	2	3	2	2	2	2	(2)	2	2	2	2	2
Electronic transactions framework	2	2	2	2	2	0	(2*)	0	2	2	2	2
Conclusion of contracts by electronic means	0	0	0	0	0	3**	0	3*	3**	3**	3**	0
E-authentication and trust services	3**	3**	3**	0	3**	3**	(3**)	4*	4**	4**	4**	3**
Facilitating paperless trade	1	1	0	3**	3**	0	(1)	0	1	3**	2	4**
Cooperation and interoperability on digital identities	0	0	0	1	2	0	0	0	2	2	2**	1
Implementation of e-invoicing systems	0	0	0	2	2	0	0	0	1	2	2	2
Regulating cross-border e-payments	0	0	0	2	3	0	0	0	1	1	0	3
Regulating cross-border logistics	0	0	0	1	0	0	0	0	0	1	0	1
Issue 2: Cross-border data flows												
Free flow of data (non-financial)	3*	3*	3*	3*	3*	3*	(3***)	0	3*	3*	3*	3*
Data localization (non-financial)	3*	3	3	3*	3*	3*	(3***)	3	3*	3*	3*	3*
Protection of personal information	2	2	2	2	3	2	(2)	2***	3	3	3	3
Free flow of financial data	3**	3**	3**	0	3**	3**	(3**)	3**	3**	3**	3**	3**
Localization of financial data	3**	3**	3**	0	3**	3**	(3***)	3**	3**	3**	3**	1
Access to government data	0	1	1	1	1	1	0	1	1	1	2	1
Supporting data innovation	0	0	0	1	1	0	0	0	1	1	0	1
Issue 3: Innovation and regulation of new technologies												
Mandatory disclosure of source code	3*	4*	4*	0	3*	4*	0	3*	3*	4*	0	4*
Regulation of ICT products that use cryptography	0	0	3*	3*	3*	3*	0	0	3*	3*	3*	3*
Adoption of standards & conformity assessment	0	0	0	0	1	0	0	0	0	1	0	1
Governance of AI and emerging technologies	0	0	0	1	2	0	0	0	(2)	1	2	1
Supply of new financial services and fintech cooperation	3**	3**	0	2	3**	3**	(1)	3**	3**	3**	3**	2
Regtech cooperation	0	0	0	0	2	0	0	0	0	1	0	0
Lawtech cooperation	0	0	0	0	0	0	0	0	0	1	0	0
Issue 4: Regulation of digital platforms												
Limiting ISP liability (non-IP)	0	3	3	0	0	0	0	0	0	0	0	0
Limiting ISP liability for IP infringement	0	4	0	0	4	3	0	0	3	3	3	0
Safety and security online	0	0	0	1	2	0	0	0	0	1	0	1
Principles on access to and use of the internet	1	1	0	1	1	2**	0	3**	1	0	1	1
Competition policy in digital markets	0	0	0	2**	1	0	0	0	1	1	1	1
Issue 5: Collective rights												
Protection of online consumers	2	2	2	2	2	2	(2)	2	2	2	2	2
Regulation of spam	2	2	2	2	2	2	(2)	3*	2	2	2	2
Cooperation on cybersecurity matters	1	1	1	1	1	0	(1)	2	1	1	1	1
Labour protections	0	0	0	0	0	0	0	0	0	1	0	0
Issue 6: Stakeholder engagement and digital inclusion												
Promoting stakeholder engagement	0	0	0	2	1	0	(1)	0	(2)	1	0	1
Facilitating digital inclusion	0	0	0	2	1	0	0	0	1	2	2	1

*Notes*: All provisions which are coded in brackets (e.g. RCEP e-commerce chapter and Australia–UK innovation chapter) reflect the fact that the provision is not subject to dispute settlement.

*Source*: Legal coding of provisions by authors (see annex for code book).

We code as ‘two’ where the commitment made is binding but non-specific. This mostly occurs where parties made a commitment to ‘cooperate’ or ‘exchange information’, or where the drafting language was vague or indeterminate, resulting in the nature of the commitment being unclear and leaving a high level of discretion to the parties with regards to implementation. For example, under ‘domestic electronic transactions framework’ in Table 1, the provisions in the Japan–US DTA are classified as ‘two’ because although they contain binding language, the commitments are unspecific, establishing that parties ‘shall maintain a legal framework governing electronic transactions consistent with the principles of the UNCITRAL Model Law on Electronic Commerce 1996’ but providing no details of what this consistency would entail. As will be further discussed below, when pertinent issues around some of the definitions and scope are not clearly provided, the provision is broad enough to allow a state to implement a wide range of approaches and avoid being in breach of its obligation, making the commitment less substantive.

Our coding of ‘three’ and ‘four’ reflects more substantive binding obligations where a state could reasonably be found in breach. Under the value of ‘three’, we put commitments that are binding and specific, with actions to be taken (or not taken) described in clear and precise language. Examples of this include Japan–US DTA and USMCA provisions on data localization, where parties specifically commit to not requiring use or establishment of computing facilities as a condition of conducting business. Finally, we assign a score of ‘four’ where a provision goes further with binding and specific language and, in addition, obligations that are more extensive in scope and very detailed. For example, Korea–Singapore's provision on paperless trade received a ‘four’ because in addition to having binding language, it also provides specific and extensive detail on what parties must do, such as the commitment to interconnect their single windows.

The second part of our coding seeks to capture the degree of public policy flexibility that parties built into each individual commitment, beyond the flexibility provided by general exceptions. As is routine practice, all the agreements we examine have general exceptions, typically based on WTO law (Article XX and Article XXI of GATT, and Article XIV of GATS), which allow governments to derogate from treaty commitments for specific public policy reasons, such as public health or national security. Many digital chapters have additional exceptions, such as the exclusion of financial services or government procurement. The EU has introduced an additional general exception in its digital trade chapters on the ‘right to regulate’ under which the parties ‘reaffirm the right to regulate within their territories to achieve legitimate policy objectives’. The provision includes a non-exclusive list of objectives to which the ‘right to regulate’ applies, mentioning several that are not explicitly stated in WTO exceptions such as climate change, and privacy and data protection.^36^ In some cases, states introduce additional flexibility by excluding the digital provisions from dispute settlement, notably in the case of the e-commerce chapter in RCEP and the innovation chapter in the Australia–UK agreement.

In addition to these agreement-wide and chapter-wide exceptions, flexibilities are built into individual provisions, which our coding aims to capture by assigning stars (*).^37^ These exceptions may narrow the scope of the obligation by listing specific sectors to which it does not apply, or they may specify qualifying criteria to justify actions that would otherwise breach the obligation.^38^ No star is assigned if no additional flexibility is built into the provision. If additional flexibility is included but the scope of the carve-out is narrow or the qualifying criteria are relatively stringent, we assign one star (*). For instance, many commitments on cross-border data flows allow for a restriction only if it is for a ‘legitimate public policy objective’, does not amount to ‘arbitrary or unjustifiable discrimination or a disguised restriction on trade’, and is not ‘greater than required to achieve the objective’. This language is borrowed from GATT Article XX, whose interpretation can sometimes be narrow.^39^

We assign two stars (**) to provisions that allow for discretionary action through expansive drafting. This may include cases where a party may breach the rule for any ‘legitimate public policy’ reason without further qualification, or where the rule is subject to a broad qualification such as ‘except as otherwise provided for in its laws and regulations’. Finally, we assign three stars (***) to exceptions that are expansively drafted and self-judging. For example, under the topic on data localization of non-financial data, we assign three stars to RCEP based on the language used in its carve-out. In particular, paragraph three of article 12.14 provides that where parties adopt measures inconsistent with the obligation ‘such measures shall not be disputed by other parties’.^40^

Combining both components of our coding methodology allows us to distinguish between provisions where the strictness of the core obligation is comparable but there are notable variations in the extent to which public policy flexibilities are incorporated, leading to differences in the overall nature of the commitment. It is worth noting that coding legal provisions requires the exercise of judgment since determining the scope of an obligation or the extent of public policy flexibilities involves interpreting intricate legal drafting. To enhance the reliability of the coding, the three authors independently coded in the initial stage and then reconciled differences, relying on existing research on digital trade and international trade law to guide final coding decisions. For more comprehensive details about the coding methodology and the issues covered, a codebook is available in the appendix.

## 3. Major Trends: A New Wave of Digital Trade Rulemaking Led by Singapore

**Figure 1.**
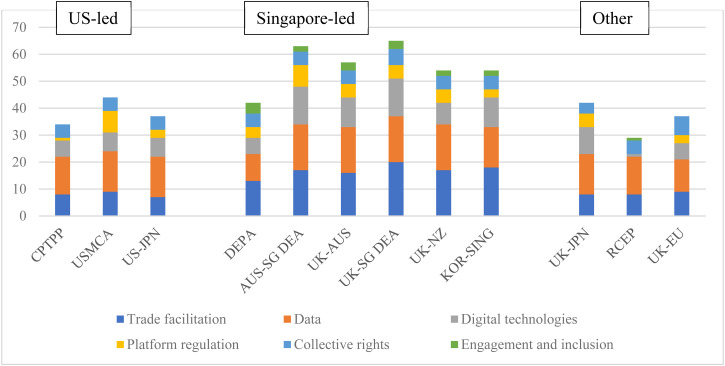
Extensiveness of commitments in recent digital trade agreements (aggregate scores) *Source:* Aggregation of legal coding of provisions by authors (see Table 1).

The detailed results of our coding exercise are provided in Table 1, which presents the agreements in chronological order according to date of signature. Figure 1 draws on our coding exercise and presents the aggregate scores for each issue area, conveying headline trends in the evolving scope of legal obligations.^41^

Our provision-by-provision analysis (Table 1) reveals substantial continuity. With the notable exception of the agreements involving China and the EU (RCEP and EU–UK agreement, respectively), recent digital agreements incorporate many core features of the US-led agreements (the first three columns of Table 1). As we explain in greater detail below, recent agreements largely replicate the provisions on cross-border data flows, data localization, source-code protection, and regulation of ICT products using encryption, where provisions in US-led agreements are substantive and binding. They also replicate provisions in areas such as consumer protection, spam, and cybersecurity cooperation, where provisions in US-led agreements are vaguely drafted or hortatory.

Alongside this continuity, we also find substantial innovation. The scope of issues covered by digital trade provisions has grown considerably since the last US-led agreement in 2019. The main innovations were introduced through the DEPA between Chile, New Zealand, and Singapore in June 2020, and the Australia–Singapore DEA in August 2020. These four states were already parties to the CPTPP, and the new agreements were used to expand the scope of digital trade topics being addressed rather than offer a radical departure from the US model. As indicated in Table 1, the DEPA and the Australia–Singapore DEA introduced provisions in 12 areas that had not been covered in the previous US-led agreements: digital identities, e-invoicing, e-payments, logistics, data innovation, standards and conformity assessment, governance of AI, regtech, safety and security online, competition in digital markets, stakeholder engagement, and digital inclusion. In the aftermath of DEPA and DEA, there was little further expansion of scope, as only two more issues were added, namely provisions on lawtech and labour protections, which were mentioned for the first time in the Singapore–UK agreement. Overall, the Australia–Singapore DEA and Singapore–UK agreements contain the most extensive commitments on digital trade (reflected in the highest aggregate scores).

Reflecting the nascent stage of national policymaking in these fast-evolving policy areas, our coding reveals that new issues are usually incorporated as soft law commitments, using non-binding or binding and vague language (assigned values of 1 and 2 in Table 1). Commitments are often broad statements of intent and agreement to cooperate, without specifying specific measures that governments will (or will not) take. This soft law approach is deliberate as it is well-suited to an emerging policy domain where technological innovation is happening apace and there is considerable uncertainty as to what ‘good’ regulation looks like, even in a domestic context.^42^ Soft law commitments encourage dialogue and coordination and aim to support the emergence of common hard law approaches over time. Indeed, there are risks in prematurely adopting a hard law approach, as we discuss below with regards to provisions on source code.

Strikingly, our provision-by-provision analysis reveals that in all 14 instances of new issues being introduced, Singapore was a party to the agreement. In addition to DEPA and the Australia–Singapore DEA, four other agreements incorporate many of the innovations: Australia–UK FTA; Singapore–UK DEA; New Zealand–UK FTA; and Korea–Singapore DEA. In the remainder of the article, we refer to these six agreements as part of a new ‘Singapore-led’ wave of digital trade agreements.

In contrast, three agreements (Japan–UK, EU–UK, and RCEP) exclude many of the Singapore-led innovations, reflected in relatively low aggregate scores (Figure 1). The timing of the Japan–UK negotiations helps explain its more limited nature. In its post-Brexit agreements, the UK pivoted away from the EU's more cautious approach to digital trade, and the Japan–UK agreement is largely modelled on the CPTPP text. It did not incorporate the novelties introduced through DEPA and the Australia–Singapore DEA, largely because it was negotiated during the same period, leaving limited time for emulation. The relatively low score of the EU–UK agreement reflects the EU's comparatively cautious approach to digital trade. Although the EU–UK agreement went beyond the EU's previous commitments^43^ the EU continues to be cautious, particularly with regards to commitments on cross-border data flows and provisions on personal data protection.^44^ RCEP similarly reflects the relatively cautious approach of China and many ASEAN countries. While it uses much of the drafting language of CPTPP, it excludes the innovations seen in the ‘Singapore-led’ agreements and, crucially, excludes the digital chapter in its entirety from dispute settlement, rendering the commitments non-enforceable (reflected in the brackets we have added to the coding of RCEP provisions in Table 1). Many RCEP commitments contain additional, extensive, and often self-judging carve-outs, as demonstrated by the high number of double and triple stars assigned to RCEP's provisions.

Beyond our text-based analysis, there is substantial primary evidence which corroborates our finding that Singapore is a norm entrepreneur in digital trade, offering a revised agenda to that found in US trade agreements. The US approach focused on boosting the competitiveness of its large technology companies in global markets by securing commitments from other governments to alter regulations perceived by these companies as impeding their expansion overseas.^45^ Priorities included prohibitions on customs duties on digital products, legal commitments securing the free flow of data across borders and prohibiting data localization, limitations on government access to source code, and limitations on the liability of internet service providers for harms arising from user-generated content and from intellectual property infringements.^46^

In contrast, Singapore is motivated by concerns that, as the digital economy evolves, governments are starting to create regulations which, if uncoordinated, create trade barriers. Through its digital trade agenda, Singapore seeks to find ways to foster regulatory alignment and build bridges across regulatory regimes.^47^ This position echoes the concerns of many states who are concerned about growing regulatory divergences between the US, EU, and China, and heightened geopolitical tensions that are leading to fragmentation, particularly in the digital economy.^48^ Singapore's aims are reflected in the contents of the digital economy agreements it has pioneered, which seek to promote the digitalization of trade as well as address regulatory fragmentation, including by promoting international standard setting and interoperability.

This agenda reflects Singapore's economic interests. As a small, trade-dependent nation, Singapore has succeeded economically by positioning itself as a critical node in global supply chains, first as a shipping hub and then as a conduit for finance and investment.^49^ Singapore has championed the digitalization of its own economy, society, and government, frequently topping global rankings^50^ and now seeks to position itself as a critical node in the global digital economy. In the words of its trade minister, Singapore is forging digital agreements so that it can ‘become one of the nodes for such data flows [in the global economy], not unlike the way we have emerged as a node for capital flow and goods flows and services flows’.^51^ Singapore's top trading partners are China and the US, so divergent regulatory approaches, particularly between these two major players, has major implications.

By articulating a different set of normative objectives for digital trade – focused on building bridges across regulatory regimes – and providing concrete examples of how these can be reflected in legal provisions, Singapore offered a new cognitive frame for thinking about the purpose and content of digital trade policy, an essential first step in a process of normative change.^52^ Singapore is also explicit about its desire to act as a norm entrepreneur. For instance, the Smart Nation strategy (2018)^53^ speaks of the government's intention to be a thought leader around technology regulation and norms. In 2019, the trade minister also set out the vision for digital trade in parliament explaining that Singapore aims to ‘advocate for an integrated, global digital economy’ and ‘co-develop international trade rules for the digital economy’.^54^

The literature highlights how normative change entails norm entrepreneurs finding platforms through which to promote their normative agenda, strategies for persuading others to adopt the norms, and allies to endorse and help socialize them. Singapore has sought to catalyse talks on digital trade at the WTO, helping set up the Joint Statement Initiative on e-commerce, which it co-chairs with Australia and Japan, and negotiate ‘pathfinder’ digital trade agreements with likeminded partners.^55^ These two platforms are complementary, multilateral negotiations that help foster dialogue and socialization, but are slowed down by divergences between the major players, while digital economy agreements reach fewer countries but are a place where innovation can occur.^56^ Moreover, DEPA was specifically designed as a promulgation mechanism. It is an open plurilateral, which other countries can join, and it comprises a set of modules that are easy for other countries to replicate and integrate into their preferential trade agreements.^57^

Alongside these rule-making initiatives, Singapore has undertaken a series of initiatives to persuade countries of the merits of its approach. These include collaborating with the WTO to hold training programmes for third countries on digital trade to foster peer-learning, and pilot projects to showcase the practical gains from implementation. Projects have involved working with Australia to trial the use of blockchain in issuing and verifying trade documents and working with the UK to trial quantum-secure cross-border electronic trade transactions.

There are signs of norm socialization and acceptance among a wider group of states. By the end of 2023, South Korea had become the first country to accede to DEPA; China, Canada, Costa Rica, and Peru had submitted applications, while others had expressed an interest in joining.^58^ As we show in this article, Singapore's innovations are spreading to agreements where it is not a signatory, including the New Zealand–UK FTA and Australia–UK FTA. Meanwhile at the WTO, by October 2023 90 members, covering 90% of global trade, had joined the WTO Joint Statement Initiative on E-commerce. Several provisions that we identify in this article as part of the ‘Singapore-led’ wave are on the WTO JSI agenda and agreement has reportedly been reached on some of them.^59^ In some cases, drafting language proposed in WTO talks emulates that found in DEPA, including by states that were not party to the agreement, such as the text on e-payments proposed by China.^60^ This suggests a growing degree of acceptance of aspects of Singapore's approach.

Moreover, there are signs that some of Singapore's ‘like-minded’ states are now acting as a ‘norm leaders’ – an actor that is quick to adopt the innovations proposed by the norm entrepreneur and works to promulgate them.^61^ This is most pronounced in the case of the UK. As we show in this article, all trade agreements that the UK negotiated after the conclusion of DEPA closely replicated the Singapore-led approach, and the UK actively seeks to promulgate this approach. As the UK's chief negotiator for the agreement with Singapore explained, ‘The challenge we set ourselves was to make the DEA the most innovative trade agreement in the world … Much of the strategic value in deals such as the DEA comes from the example they can set to others … The UK–Singapore DEA is set to become not only the new gold standard for the UK's digital trade agreements but also, we hope, a modern trade agreement that others can build on.’^62^ Australia also seems to be playing a norm leadership role. Although it has pursued fewer digital trade agreements than the UK, it is active in WTO negotiations and initiatives within ASEAN. In contrast, while Chile and New Zealand helped to forge the Singapore-led approach, reflected in the DEPA agreement, we found no evidence that they are acting as norm leaders.

Overall, our analysis reveals substantial innovation in digital trade rulemaking, led by Singapore. We have found evidence of Singapore acting as a norm entrepreneur, and other countries, including the UK, acting as norm leaders. The norms and rules encapsulated in this ‘Singapore led’ approach are gradually being institutionalized in rules and organizations and some degree of socialization has occurred. However, the innovations embodied in the Singapore-led wave are yet to be widely accepted as global norms for digital trade governance.

## 4. The New Wave of Digital Rulemaking: In-Depth Analysis of Issue Areas

This section delves into the details of the six agreements we identify as part of the ‘Singapore-led’ wave of digital trade agreements, seeking to establish in precise terms how they compare with the previous US-led wave. The analysis is structured around the issues and scores presented in Table 1, focusing on areas where difference between the US-led and Singapore-led approaches are most prominent.^63^

### 4.1 Trade Facilitation

The provisions on trade facilitation feature prominently in recent digital trade agreements, accounting for nine of the 34 topics in our database. The aim of these provisions is to support the digitalization of supply chains as, at present, end-to-end digital trade remains rare owing to non-interoperable systems, misaligned regulations, and inadequate underlying legislation.

In several areas, notably commitments on a moratorium on custom duties on electronic transmissions, electronic transaction frameworks, and electronic authentication and trust services, the Singapore-led agreements largely replicate the drafting language found in US-led agreements.^64^ One notable difference is that commitments on electronic transactions frameworks are updated to refer to the UNCITRAL Model Law on Electronic Transferable Records (2017), although commitments to adopt the model law remain non-binding. A cross-border transaction requires, on average, exchange of 36 documents and 240 copies, and less than 1% of trade documents are fully digitized.^65^ The 2017 model law seeks to enable the legal use of electronic transferable records that are linked to the delivery of goods or payment of money, both domestically and across borders, including bills of exchange, promissory notes, consignment notes, bills of lading, and warehouse receipts.

Singapore was the second country in the world to adopt the UNCITRAL 2017 model law after Bahrain and, in addition to promoting its adoption through commitments in its digital trade agreements, it has also embarked on pilot initiatives with Bahrain and the UK to explore how best to implement the legal changes.^66^ Since 2021, the UK has also sought to champion the model law, drafting domestic legislation and promoting the model law during its G7 presidency. Another interesting feature is that the agreements that the UK is a party to, all include specific additional commitments on the conclusion of contracts by electronic means, modelled on language from EU agreements, to which the UK was a party prior to Brexit.

The novel aspects of the Singapore-led agreements are in the areas of paperless trade, digital identities, e-invoicing, e-payments, and logistics. While the commitments examined show a desire to improve regulatory cohesion and interoperability, they are mostly non-binding or include soft commitments to cooperate, indicating that specific binding approaches in these aspects of digital trade facilitation are yet to emerge.

Commitments on ‘paperless trade’ aim to support the digitalization of transactions between private actors and governments and government-to-government (e.g. digital single windows). US-led agreements include short, non-binding commitments to make trade administration documents available in electronic form and accept electronically submitted documents as legally equivalent to paper versions.^67^ In contrast, the Singapore-led agreements introduce much more extensive commitments. In a lengthy provision, the parties in DEPA commit *inter alia* to: making electronic versions of all trade administration documents publicly available and endeavouring to provide them in machine-readable format; accepting electronic versions of trade administration documents as the legal equivalent of paper documents; establishing a single window and endeavouring to ‘establish or maintain a seamless, trusted, high-availability, and secure interconnection of their respective single windows’; and endeavouring to develop compatible and interoperable data exchange systems, and promoting and advancing the use of such systems.^68^ This language is largely replicated in the Australia–Singapore DEA, Singapore–UK DEA, and Korea–Singapore DEA. The Korea–Singapore DEA includes an additional binding commitment to interconnect the parties' single windows.^69^ The New Zealand–UK FTA, stops short of a binding commitment to accept electronic documents as legally equivalent to paper versions,^70^ while the Australia–UK FTA simply makes best-endeavour commitments, like the CPTPP. Singapore's moves to promote interconnectivity of single windows and interoperable data exchange systems reflects its wider digitalization agenda. Singapore was a very early adopter of a national single window and has since embarked on a series of projects to achieve interconnectivity, including with Hong Kong, part of a wider move to promote a ‘Global Trade Connectivity Network’.^71^

Provisions on digital identities were introduced for the first time in the DEPA and aim to promote regulatory coherence and, ultimately, enable mutual recognition and interoperability, making it easier to use digital identities in cross-border transactions. In DEPA, the parties ‘endeavour to promote the interoperability between their respective regimes for digital identities’,^72^ and similar non-binding wording is found in the Korea–Singapore DEA. The Australia–Singapore DEA contains a slightly stronger commitment as the parties ‘shall pursue the development of mechanisms to promote compatibility between their respective digital identity regimes’,^73^ a commitment mirrored in the Australia–UK FTA, New Zealand–UK FTA, and Singapore–UK DEA. Some agreements are accompanied by Memorandums of Understanding (MOUs) under which they agree to collaborate to work together on digital identities including developing a roadmap for interoperability and mutual recognition.^74^ The relatively soft language in the Singapore-led wave of agreements reflects the fact that countries are at different stages in implementing digital identity regimes, and have very different approaches, ranging from national centralized digital identity schemes to decentralized models based on certification.

Provisions on e-invoicing and e-payments were also introduced through DEPA. E-invoicing schemes enable the exchange of invoice data directly between suppliers' and buyers' financial systems, using a pre-defined set of common data standards or structures, removing the need for paper invoices and manual processing along supply chains. DEPA requires parties to ensure that e-invoicing measures support cross-border interoperability and are based on international standards, where they exist, and to share best practices and collaborate on promoting the adoption of e-invoicing.^75^ Similar wording is found in the Australia–Singapore DEA, Singapore–UK DEA, New Zealand–UK FTA, and Australia–UK FTA, while in the Korea–Singapore DEA the provision is entirely non-binding.

International payments are indispensable for cross-border trade and investment, but cross-border payments are still slow and expensive, with only around 60 countries having instant payment systems for domestic bank accounts in 2021.^76^ DEPA includes a general binding commitment to foster the adoption of internationally accepted standards, interoperability, and innovation and competition in cross-border electronic payments.^77^ A list of possible actions is included, couched in non-binding language. Similar commitments are found in the Australia–UK FTA and Singapore–UK DEA (in entirely non-binding language) but absent from the New Zealand–UK FTA. The commitments in the Australia–Singapore DEA and Korea–Singapore FTA are more substantial than those in DEPA. This includes a pledge not to unjustifiably discriminate between financial and non-financial institutions in terms of access to electronic payment systems, and to promote the adoption of international standards to enable greater interoperability between electronic payment systems.^78^ Again, this variation reflects the state of play on the ground, as the central bank of Singapore has pioneered cross-border e-payment collaborations with central banks in several countries, including Malaysia, India, Thailand, and Australia.

**Figure 2.**
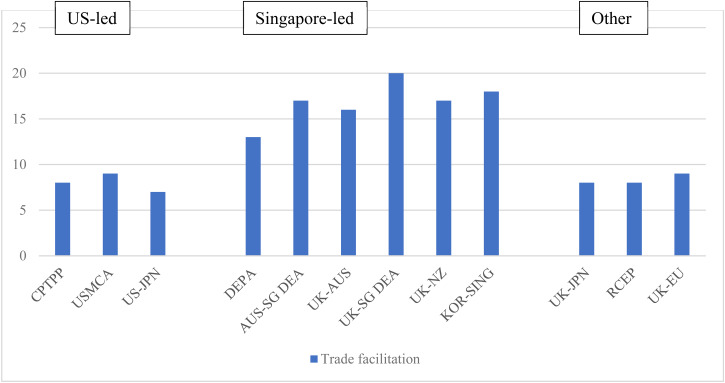
Extensiveness of trade facilitation commitments *Source:* Aggregation of legal coding of provisions by authors (see Table 1).

Logistics is the final area within trade facilitation where the Singapore-led agreements introduce changes. Last-mile delivery has proven to be one of the most challenging aspects of the shift to online retail sales, with logistics companies struggling to keep up with demand; retailers having little control or oversight of delivery companies; and delivery vehicles causing additional traffic congestion and pollution. In response, businesses are experimenting with on-demand and dynamic routing solutions, drones, and parcel lockers. These developments raise new questions for governments about how best to regulate these new delivery and business models.^79^ DEPA was the first agreement to include a commitment on logistics, although it simply commits the parties to ‘endeavour to share best practices and general information’. Such commitments remain uncommon, as the Singapore–UK DEA and Korea–Singapore DEA are the only other agreements to include a similar, non-binding provision (Figure 2).

### 4.2 Data Governance

Data are fundamental to the global digital economy, and data flows underpin digital trade. The volume of data crossing borders has grown exponentially. While it is hard to gauge the economic value of these flows, some argue that cross-border data flows are at least as valuable as trade in goods.^80^ Governments have different regulatory priorities, ranging from promoting business, to protecting privacy of citizens, to pursuing national security objectives, leading to very different approaches to data regulation.^81^ Divergences are a major source of trade frictions, including among the US, EU, and China, and differences are reflected in very different commitments in their trade agreements.^82^

The Singapore-led agreements replicate the US-led approach to commitments on cross-border data flows, data localization, and personal data protection, using the drafting language of the CPTPP with very minor modifications. This said, there are some subtle differences in specific agreements that are worth noting.

Financial data are usually subject to separate provisions, usually found in the financial services chapters of FTAs, so stand-alone digital economy agreements may leave out commitments on financial data altogether (as is the case with DEPA) or include separate provisions for financial data. Reflecting the sensitivities around financial data, including for prudential reasons, commitments are routinely subject to more extensive exceptions (reflected in the higher number of stars in Table 1). While commitments in the Singapore-led agreements are modelled on the CPTPP, the one exception is the Korea–Singapore agreement, where the commitment on localization of financial data is couched in non-binding language, reflecting Korea's preference for retaining a high level of regulatory autonomy.^83^

The drafting language on personal data protection is also slightly different to the US-led agreements. The US-led agreements have binding but vaguely worded commitments. In the CPTPP, parties commit to adopting or maintaining a legal framework that protects the personal information of users of electronic commerce, while ‘taking into account relevant international guidelines’,^84^ and the USMCA specifically references the OECD Guidelines on Privacy and Trans-border Flows of Personal Data and lists key principles on data protection. Such provisions are supplemented with a note that a party can comply with this obligation by adopting measures ranging from comprehensive privacy or personal data protection laws to voluntary privacy undertakings by companies.^85^

By recognizing voluntary undertakings by private companies as sufficient for safeguarding personal data protection, US-led agreements set a very low bar for personal data protection measures. In addition, by recognizing voluntary undertakings as sufficient and simultaneously requiring (under the data flow commitments) that any measure imposed on data flows ‘does not impose restrictions on transfers of information greater than are required to achieve the objective’, US-led agreements effectively impose a ceiling on the stringency of personal data protection measures.^86^ Moreover, if a party to US-style commitments adopts a comprehensive approach to data protection (such as the EU's GDPR) it may be open to challenge by its trading partner on the grounds that its approach is more burdensome than necessary, as it could have met its obligations through a less burdensome approach based on voluntary undertakings.^87^

The Singapore-led agreements replicate the US-led approach, with some modifications. In DEPA there is a binding commitment to ‘adopt non-discriminatory practices in protecting users of electronic commerce from personal information protection violations occurring within its jurisdiction’^88^ (under US-led agreements the wording is non-binding). Similarly, the commitment in DEPA on promoting compatibility and interoperability between respective regimes for protecting personal information is also stronger than in US-led agreements, where it is couched in best-endeavour language. It is noteworthy that reference to ‘voluntary undertakings’ is dropped from the note in the New Zealand–UK FTA and the Singapore–UK DEA.

US-led agreements introduced commitments on ‘open government data’, which are largely replicated in the Singapore-led agreements. Proponents argue that by making their datasets publicly available, public institutions increase their transparency and accountability to citizens, while the ability to use, reuse, and freely distribute datasets promotes business creation and innovative, citizen-centred services.^89^ Since the Japan–US DTA and USMCA, digital trade agreements have usually included provisions on open government data, although these are generally worded as best endeavour commitments to digitalize the data that governments already make public, rather than commitments to further increase the availability of government data.^90^ DEPA goes further by identifying specific areas of possible cooperation^91^ while the New Zealand–UK FTA contains binding language, although it only obliges, for governments to provide ‘the opportunity to request’ the disclosure of specific government data and information.^92^

One criticism of open government data is that major technology companies benefit disproportionately as they have the capacity to collect open data and correlate it with the ‘closed data’ they hold.^93^ There is a growing concern that major technology companies leverage their control over data to reinforce their market power, prompting some jurisdictions to experiment with legally mandated data sharing.^94^ DEPA was the first trade agreement to make reference to ‘data sharing’ although the commitments are generally worded and non-binding. In DEPA, the parties recognize that cross-border data flows and data sharing ‘enable data-driven’ innovation, and acknowledge that data sharing mechanisms, such as trusted data sharing frameworks and open licensing agreements, aid in innovation and knowledge diffusion, and foster competition. The parties commit to ‘endeavour to cooperate’ on data sharing projects and mechanisms.^95^ Similar provisions are found in the Australia–Singapore DEA, Australia–UK FTA, Singapore–UK DEA, and Korea–Singapore DEA.

The approach to data governance in the EU–UK TCA and RCEP are very different. Although the EU–UK TCA exceeds the commitments on data flows and data localization found in previous EU agreements, the commitments remain far less extensive than those found in US-led and Singapore-led agreements. In the EU–UK TCA, the EU declined to make a general commitment on cross-border data flows although it did agree to a list of specific prohibitions related to data localization.^96^ Several scholars have observed that the narrow exception in US-led agreements may unreasonably limit the scope of government measures intended to promote other significant policy goals, including personal data protection.^97^ The EU, in particular, faces difficulties in making any legal commitment that would compromise its ability to implement measures related to personal data protection and the high data protection standards of its General Data Protection Regulation (GDPR).^98^ The EU–UK TCA also includes comprehensive carve-out provision that allows for a very high level of regulatory autonomy with regard to personal data protection (attracting three stars in our coding). The parties agree that nothing in the agreement can prevent a party from adopting or maintaining measures to protect personal data and privacy, including cross-border data transfers, as long as the party's law provides for instruments enabling transfers under conditions of general application for the data's protection. The term ‘conditions of general application’ refers to objective conditions that apply broadly to many economic operators, covering various situations and cases, implying that the EU can adopt any approach it sees fit so long as it does so through a general rather than a specific (discriminatory) approach.^99^

**Figure 3.**
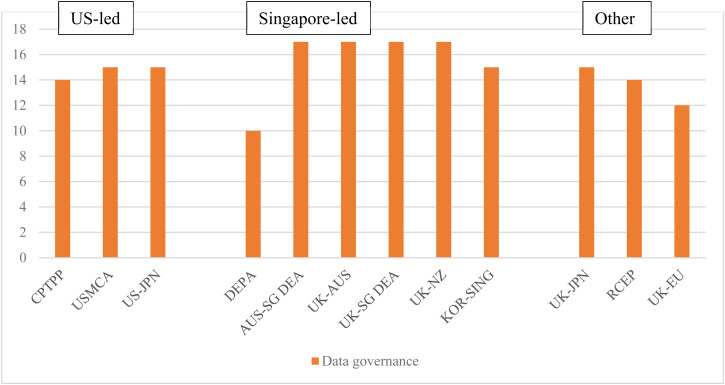
Extensiveness of data governance commitments *Source:* Aggregation of legal coding of provisions by authors (see Table 1).

Reflecting on the sensitivities of some RCEP members, notably China, and determination to preserve regulatory autonomy over data, RCEP adopts the general CPTPP approach but builds in extensive self-judging exceptions. In the area of cross-border data flows, a party can implement an inconsistent measure ‘*that it considers necessary* to achieve a legitimate public policy objective’ or ‘*that it considers necessary* for the protection of its essential security interests’ [emphasis added]. Moreover, in the case of essential security, ‘such measures shall not be disputed by other Parties’ (Figure 3).^100^

### 4.3 Regulation of Digital Technologies

Governments are increasingly concerned about the risks associated with the widespread use of digital technologies, such as algorithms and cryptography, including discrimination and a lack of fairness and accountability.^101^ Trade agreements have begun to include provisions regulating the use and access of digital technologies, which have an impact on the fundamental rights of individuals, various government interests (such as law enforcement and innovation), and the commercial interests of technology firms.^102^ As such, finding the delicate balance between the multiple interest at stake is a challenge that trade negotiators, like domestic regulators, are now faced with.

In the area of regulation of digital technologies, the Singapore-led wave of agreements largely replicate the US-led commitments on the mandatory disclosure of source code, regulation of ICT products that use cryptography, and regulation of new financial services, with minor modifications. However, they also innovate by adding new commitments in four areas: digital standards and conformity assessment, AI governance, regtech, and lawtech.

Provisions banning measures that mandate access to, or transfer, or source code have been at the heart of US-led agreements. They aim to protect technology firms from government measures requiring trade secrets to be disclosed as a prerequisite for operating in certain industries, most notably in China. US-led trade agreements offer extensive and binding intellectual property protection for source code which goes beyond the level of protection afforded under the WTO Trade-Related Aspects of Intellectual Property Rights (TRIPS) Agreement, with some deals explicitly including algorithms within the scope of protection. For instance, the CPTPP refers to the ‘source code of software’,^103^ while the USMCA explicitly protects algorithms expressed in source code.^104^ Referring specifically to ‘algorithms expressed in source code’ provides a higher level of protection by clarifying the scope of the provision, and this language has been criticized for potentially conflicting with proposals to regulate algorithmic decision-making, including addressing the risk of discrimination.^105^ Prohibitions on disclosure of source code, software, and algorithms can also impede access to training datasets, hinder technology access and market competition, and limit the availability of open-source software.^106^ The source code provisions in US-led agreements provide some exceptions, although they are narrow and do not address all public policy concerns.^107^ The CPTPP only allows an exception to modify source code to comply with laws and regulations, while the USMCA and Japan–US agreements provide even narrower exceptions, granting access only to selected bodies on a case-by-case basis, thereby leaving no room for *ex-ante* regulation and oversight.^108^

The Singapore-led wave of agreements includes source code provisions modelled on the provisions in US-led agreements. The text of Australia–Singapore, Australia–UK, and EU–UK agreements includes provisions on the source code of software, but does not include specific mentions to algorithms. In contrast, Singapore–UK, Japan–UK, and Korea–Singapore agreements include a direct reference to the ‘algorithm expressed in that source code’. The notable exceptions are the ones to which New Zealand is a party (DEPA and the New Zealand–UK FTA) and RCEP, which do not include such provisions. In November 2021, the Waitangi Tribunal found the source code provision in the CPTPP to be in breach of the Treaty of Waitangi, with Māori tech experts highlighting the risks of biased assumptions in algorithmic design or training data.^109^ Following this ruling, New Zealand has excluded provisions on source code disclosure from its trade agreements. The absence of similar provisions in RCEP is not surprising given China's history of requiring technology transfers as a condition of doing business.^110^

While there has been some evolution and change in the drafting language of the exceptions when compared to that of a US-led agreement, the level of public policy flexibility granted to the parties in Singapore-led agreements is still limited. The Australia–Singapore agreement widens the range of authorities that can request parties to preserve and make available the source code, including government agencies and requests made in the context of administrative proceedings in the list of exceptions.^111^ The Japan–UK text includes a mention of conformity assessment bodies, and the EU–UK TCA includes exceptions for measures adopted in the context of a ‘certification procedure’.^112^ Notably, the EU–UK agreement expands the scope of the exception by explicitly stating that the provisions do not affect requirements related to competition law, the ‘protection of public safety with regards to users online’, and intellectual property rights.^113^

Subsequent deals include a mix of this wording. The Australia–UK deal allows more government bodies to require disclosure, including a government agency, regulatory body, administrative tribunal, or judicial authority, or a designated conformity assessment body.^114^ The Singapore–UK DEA does not mention government agencies, but also includes a mention of conformity assessment bodies and allows access in a wider range of contexts (including for monitoring compliance with codes of conduct and standards).^115^ The Korea–Singapore text includes a reference to government agencies' administrative proceedings, but no reference to conformity bodies.^116^ Overall, while provisions in the Singapore-led texts are slightly broader exceptions when compared to the US-led texts, these exceptions do not constitute a comprehensive public policy carve-out and are not sufficient to ensure governments will have flexibility to implement *ex ante* regulation aimed at improving algorithmic transparency and accountability, such as transparency requirements being introduced with the AI Act in the EU. Further, by focusing only on public authorities, these exceptions side-line technical experts from academia and civil society.^117^ Importantly, they may not guarantee access for individuals, workers, or businesses concerned about potential unfair treatment linked to algorithmic decision-making.^118^

The Japan–US agreement was the first to include provision on the use of ICT products that use cryptography, and this provision was replicated in all of the more recent agreements we examined, with the exception of RCEP and the EU–UK TCA.^119^ Overall, these provisions are very similar and prohibit parties from adopting regulation that requires access to a particular cryptography technology, production process, or information – for example the access key.^120^ However, while encryption of private communications is crucial in safeguarding the privacy and freedom of speech of individuals,^121^ businesses might also use it to evade scrutiny of their practices, which could result in reduced transparency and accountability of their operations. Therefore, there is a need for trade provisions to strike a balance when safeguarding the interests served by cryptographic technology.

Except for the Japan–US DTA (which does not cover services liberalization), US-led agreements include commitments on the supply of new financial services that promote market access for Fintech companies. Under the CPTPP for instance, the parties commit to ‘permit a financial institution of another Party to supply a new financial service that the Party would permit its own financial institutions, in like circumstances, to supply without adopting a law or modifying an existing law’,^122^ with a ‘new financial service defined as “a financial service not supplied in the Party's territory that is supplied within the territory of another Party’. This provision enables Fintech innovators to register and develop their project in the territory of any of the signing parties and be treated as an innovator, including having access to regulatory sandboxes, where the regulatory regime is softened to allow for the creation and testing of new financial services.^123^ However, this commitment is qualified by an exception specifying that ‘a Party may determine the institutional and juridical form through which the new financial service may be supplied and may require authorization’, although an authorization decision must be made ‘within a reasonable period of time’. The Party may refuse the authorization but ‘only for prudential reasons’.^124^

The Singapore-led agreements typically include commitments on providing market access for new financial services that replicate the US approach (although these are absent from DEPA and the Korea–Singapore agreement). Several also include a commitment to promote cooperation with, for instance, the Singapore–UK DEA stating that the Parties ‘shall endeavour to collaborate, share knowledge, experiences and developments’ to advance financial integrity, consumer protection, financial inclusion, financial stability, operational resilience, sustainability and facilitation of cross-border development of new financial services (Singapore–UK DEA, article 8.53). Similar language is found in the Australia–Singapore DEA and the New–Zealand–UK FTA. DEPA, which does not cover financial services, excludes a commitment on the supply of new financial services and only includes a binding commitment to cooperate in the promotion and regulation of financial innovation. An MOU on a ‘fintech bridge’ accompanies the Singapore–UK DEA, and aims at reviving an earlier cooperation agreement, as well as strengthening cooperation between regulators and industry to promote the sector.

In terms of new issues, Singapore-led agreements introduced commitments to promote the development of standards and conformity assessment for digital technologies. US-led agreements include general commitments to build on WTO agreements around standards, trade facilitation, and tariff reductions, but they are not tailored for digital trade.^125^ Specific commitments appear in three agreements (Australia–Singapore DEA, Singapore–UK DEA, and Korea–Singapore FTA).^126^ In the new provision, the parties recognize the importance of standards, technical regulations, and conformity assessment procedures in fostering the digital economy and reducing barriers to trade. They make non-binding commitments to cooperate in developing and adopting standards that support digital trade and to ‘endeavour to share information’. Although these provisions are mainly aspirational, the Korea–Singapore deal provides more detailed language, listing principles and procedures that could be observed when developing standards for the digital economy.^127^

All six of the Singapore-led agreements incorporate provisions that reflect the growing interest in regulating AI, which were absent from US-led agreements.^128^ However, these are mostly procedural obligations or weak commitments to cooperate, rather than substantive binding commitments, and in some cases are accompanied by MOUs. Some agreements, such as DEPA and the Australia–Singapore DEA, focus on ethical governance frameworks and promote the adoption of principles for the responsible use of AI.^129^ The Australia–UK deal is more focused on reaping the economic benefits of these technologies but also recognizes the relevance of governance frameworks.^130^ The Singapore–UK DEA features a more balanced provision, including substantive obligations to develop policy frameworks and promote algorithmic transparency,^131^ while the New Zealand–UK agreement establishes cooperation on matters such as ethical use and industry-led standards to mitigate issues such as unintended biases and existing divides.^132^ As in the Singapore–UK deal, the New Zealand–UK article refers to measures to improve fairness and accountability of AI, but provides a slightly improved wording of the provision, establishing that cooperation may include ‘ethical use, industry-led standards, and algorithmic transparency, to address issues such as unintended biases and exacerbation of existing divides, by ensuring human diversity is recognised in the development of technologies’.^133^ While many governments are grappling with AI regulation, Singapore has been particularly active in seeking to shape the global policy agenda, launching its Model AI Governance Framework at the World Economic Forum in Davos in 2019, and launching the world's first testing framework and toolkit ‘AI Verify’ in 2022. This was followed by the AI Verify Foundation in 2023, a related multi-stakeholder organization where members can collaborate on AI governance and testing, and which aims to shape international standards on AI.

**Figure 4.**
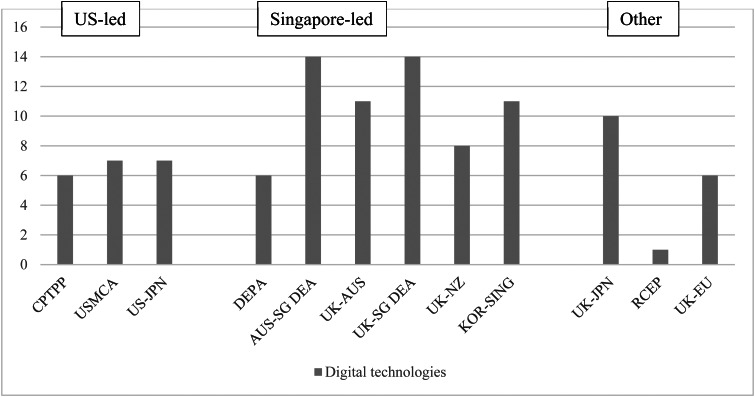
Extensiveness of commitments on regulation of digital technologies *Source:* Aggregation of legal coding of provisions by authors (see Table 1).

Provisions on regtech and lawtech were not included in the US-led wave of agreements and feature in only two of the more recent agreements (Australia–Singapore DEA and Singapore–UK DEA). These provisions encourage the adoption of digital technologies in the legal and regulatory sectors, with the aim of improving efficiency, reducing costs, and increasing regulatory compliance. Lawtech provisions typically focus on promoting digital technologies to improve the efficiency and accessibility of legal services, for example through online dispute resolution mechanisms.^134^ Regtech provisions, in turn, are focused on promoting the use of digital technologies to improve regulatory compliance and reduce the burden of compliance for businesses, usually with a focus on financial services (and are usually bundled with commitments on fintech).^135^ The Australia–Singapore DEA was the first to refer to regtech and commits the parties to encourage collaboration between regulators and agencies and promote industry collaboration.^136^ The more recent Singapore–UK DEA provides similar (although non-binding) language on regtech collaboration,^137^ and innovates by including the first stand-alone provision on lawtech.^138^ The article lists potential areas for collaboration, including establishing a dialogue; encouraging the sharing of knowledge between their respective regulators, academics, representative bodies, and industry bodies; and encouraging service suppliers to explore new business opportunities in the other party's territory (Figure 4).

### 4.4 Regulation of Internet Platforms

The growing penetration of digital platforms creates a new dimension of regulatory and public policy concerns, given the types of content internet users are exposed to and to the interconnected structure of digital ecosystems.^139^ Notably, there has been growing concern about online safety and the extent to which technology companies should take measures to protect users from online harm, which has grown alongside the increasing market power of digital platforms,^140^ and efforts to promote innovation and competition in digital markets.^141^

US-led agreements focused on protecting internet platforms from liability for third-party generated content, protections that are seen as crucial for the growth of online platforms and services.^142^ Internet platform liability provisions have been specific and binding, establishing prescriptive rules on the type of liability regime parties should adopt, while providing no policy or regulatory flexibility through carve-outs or exceptions. During TPP negotiations, the US introduced a commitment aimed at limiting the liability of internet platforms with regard to infringement of intellectual property, a provision that was then suspended when the US withdrew.^143^ The USMCA^144^ and the Japan–US DTA^145^ agreements went further, including provisions on general intermediary liability explicitly modelled on the contentious section 230 of the US Communications Decency Act (CDA), which provides a blanket liability waiver for internet companies that host user-generated content as regards the behaviour of their users.^146^ In both cases, there are no carve-outs or flexibilities that would allow parties to diverge from the safe-harbour model adopted by section 230 of the CDA, exporting the controversial US framework to its trade partners.^147^ The USMCA, in particular, also includes incredibly detailed and binding rules on intermediary liability for intellectual property infringement, committing parties to adopt rules that mimic section 512 of the US Digital Millennium Copyright Act (DMCA).^148^ Except for DEPA and Korea–Singapore DEA, which do not include any provisions on this matter, the Singapore-led agreements follow the original TPP language and only include provisions limiting the liability of internet platforms with regards to infringement of intellectual property. These provisions are generally considered less controversial when compared to broader liability rules, as several countries, including the US and EU, have enacted domestic legislation that protects internet service providers from copyright liability under safe harbour rules.^149^ Notably, the influential US model has also significantly influenced copyright law and policy in Singapore.^150^

Four of the agreements we classify as ‘Singapore-led’ include novel commitments aimed at enhancing internet safety and trust.^151^ This reflects a broader trend in which governments worldwide are discussing domestic regulation. This is for the purpose of increasing the accountability of internet platforms for the content they host, and to prevent the spread of harmful content, such as hate speech, disinformation, and violent extremist content that can harm individuals and the overall health of the digital public sphere.^152^ However, the commitments are mostly procedural and non-binding. For instance, DEPA contains provisions recognizing the relevance of safety and security online for supporting the digital economy and the importance of a multi-stakeholder approach, but only commits parties to ‘endeavour to cooperate’ in advancing global solutions.^153^ Among the agreements examined, the Australia–Singapore DEA features the strongest language on safety and security, with commitments to create and promote a safe online environment, protect users from harmful content, and collaborate within international forums.^154^ While these commitments appear in the agreements to which Singapore is a party, they are absent from the Australia–UK and New Zealand–UK agreements, suggesting that this is a commitment which is yet to be emulated.

US-led agreements, including the CPTPP^155^ and USMCA,^156^ do not include strong provisions on internet access, merely acknowledging the benefits of user choice in accessing services and applications, with a broad mention of ‘reasonable network management’.^157^ This aligns with the US government's domestic policy at the time of revoking network neutrality commitments.^158^ Singapore-led agreements, including DEPA,^159^ the Australia–Singapore DEA,^160^ and the Korea–Singapore deal,^161^ mirror this weak approach, simply ‘recognizing the benefits’ of an open internet. In contrast, EU-led texts include stronger commitments. The Japan–UK agreement requires parties to adopt or maintain appropriate measures to ensure consumers' access to the internet, ‘subject to reasonable, transparent and non-discriminatory network management’.^162^ The EU–UK TCA features the most specific provision, commitment parties to ensure users' access to services and applications, subject to network management that is not only reasonable, transparent, and non-discriminatory, but also adding the term ‘proportionate’.^163^ However, in both the Japan–UK and the EU–UK agreements, there are wide carve-outs in place, as commitments are subject to the parties' ‘applicable policies, laws and regulations’.

**Figure 5.**
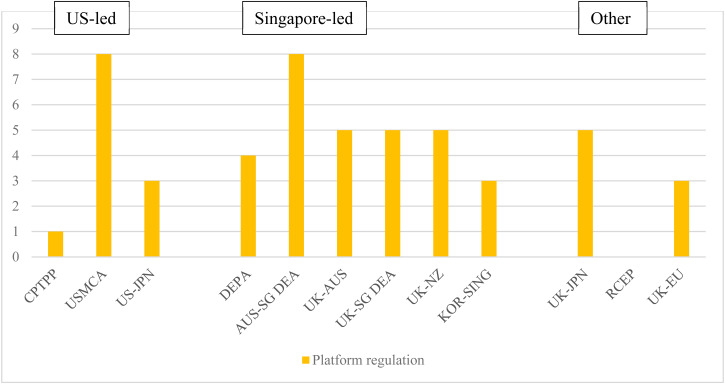
Extensiveness of commitments on regulation of internet platforms *Source:* Aggregation of legal coding of provisions by authors (see Table 1).

Competition in digital markets is another area where provisions are starting to emerge, with language first introduced through DEPA. The need to enhance competition in digital markets has been at the forefront of the agendas of policymakers and regulators, as there is a growing understanding that conventional competition laws need updating to address the unique challenges of digital markets.^164^ While there are variations in wording, the Australia–Singapore DEA,^165^ Australia–UK FTA,^166^ Singapore–UK DEA,^167^ and New Zealand–UK deal^168^ contain similar non-binding commitments expressing specific competition concerns related to digital markets. These provisions acknowledge the significance of international cooperation and outline a non-exhaustive list of areas where parties may collaborate, including exchanging information, sharing best practices, providing advice or training, and exchanging officials. The language in DEPA is slightly stronger, as the parties have agreed to cooperate on competition enforcement issues in digital markets. However, this commitment is subject to the carve-out that parties ‘shall cooperate in a manner compatible with their respective laws, regulations and important interests, and within their reasonably available resources’ (Figure 5).^169^

### 4.5 Collective Rights

The rise of the digital economy has brought with it distinctive challenges for consumer protection, labour rights, and cybersecurity in the context of international trade. Global consumers are often hesitant to engage in cross-border transactions online, owing to a lack of security and insufficient mechanisms for addressing digital-specific issues. While some trade agreements have made progress in regulating consumer issues, emerging issues such as spam, cybersecurity, and labour protection have traditionally been perceived as non-trade related, leading to slow progress.^170^ However, there is a growing recognition that a well-functioning digital economy must protect consumers, workers, and the digital infrastructure.

US-led agreements included commitments to protect online consumers, regulate spam, and cooperate on cybersecurity measures. These have been largely replicated in the Singapore-led agreements, although they have also been extended in some instances. Provisions on online consumer protection in US-led agreements oblige parties to ‘adopt or maintain consumer protection laws to proscribe fraudulent and deceptive commercial activities that cause harm or potential harm to consumers engaged in online commercial activities’.^171^ The CPTPP and USMCA also contain commitments to cooperate to enhance consumer welfare in the context of digital trade, cross-referencing the commitments made on consumer protection elsewhere in the agreement so that they also apply to an online context.

The Singapore-led agreements generally follow the approach of US-led agreements with some adjustments. Notably DEPA goes slightly further than the US-led agreements as the parties ‘recognize the importance of’ providing redress for online consumers, including when they transact with suppliers from another party, and the parties will ‘endeavour to explore’ the benefits of mechanisms, including alternative dispute resolution, to facilitate the resolution of claims relating to e-commerce transactions.^172^ Similar language is found in the other Singapore-led agreements. The one exception is the New Zealand–UK agreement, which takes a different approach, inserting a provision in its consumer protection chapter that commits the parties to ‘provide consumers engaged in online commercial activities with a level of protection not less than that provided under its law to consumers engaged in other forms of commerce’.^173^ The consumer protection chapter also includes a binding commitment on facilitating access to cross-border redress which is not found in the other agreements we examined, providing a higher level of online consumer protection.^174^

Provisions on spam in the US-led agreements are binding and vague, requiring parties to take measures to minimize unsolicited electronic messages and provide recourse against suppliers that do not comply, but providing governments with latitude to determine how they do this. USMCA extends the scope to ‘unsolicited commercial electronic communications sent other than to an electronic mail address’.^175^ However, these commitments have been criticized as they do not address current concerns such as privacy infringement, due process issues, and the increase of spam attacks through botnets and malware.^176^ Singapore-led agreements follow the same approach as the US. In agreements where the UK is a party, an additional clause is included, drawn from EU agreements. It states that ‘Each Party shall ensure that commercial electronic messages are clearly identifiable as such, clearly disclose on whose behalf they are made, and contain the necessary information to enable recipients to request cessation free of charge and at any time.’^177^ The approach of Singapore-led agreements continues to be less robust than that of the EU, where commitments have a wider scope as they apply to ‘unsolicited direct marketing communications’ and prior consent is required, except in case of advertising the supply of ‘their own similar goods and services’.^178^

Cybersecurity has become an increasing concern, with consumer and business confidence in the digital economy undermined by a range of cyber-attacks including hacking, phishing, malicious software, and distributed denial of service against websites. US-led trade agreements include non-binding commitments to cooperate in cybersecurity. The CPTPP commitment is very general, simply stating that the parties ‘recognize the importance of’ building the capabilities of their national entities responsible for computer security incident response; and using existing collaboration mechanisms to cooperate to identify and mitigate malicious intrusions or dissemination of malicious codes that affect the electronic networks of the parties’.^179^ The wording in USMCA and Japan–US DTA is more extensive, although it remains non-binding. In particular, these agreements advocate risk-based approaches in addressing cyber threats.^180^

Singapore-led agreements follow the US approach in making non-binding commitments, although there are slight variations in the drafting. DEPA, Australia–Singapore DEA, and Korea–Singapore follow the CPTPP text, adding a clause on the importance of workforce development in the area of cybersecurity. The Singapore-led agreements to which the UK is a party have more extensive language, although it remains non-binding. The Australia–UK FTA text follows the USMCA text, making reference to risk-based approaches, and also includes the DEPA reference to workforce development, and a best-endeavour commitment to dialogue between the parties. The Singapore–UK DEA text makes further additions, including reference to establishing mutual recognition of a baseline security standard for consumer Internet of Things devices, although again, it stops short of making a binding commitment.^181^ The EU–UK TCA is the only agreement we reviewed that contains a binding commitment, although it remains a very general commitment to cooperate to (EU–UK TCA article 704). Mishra criticizes the non-binding nature of such commitments for their narrow focus, arguing that these agreements miss the opportunity to include multi-stakeholder and industry mechanisms for standard-setting on cybersecurity issues.^182^

**Figure 6.**
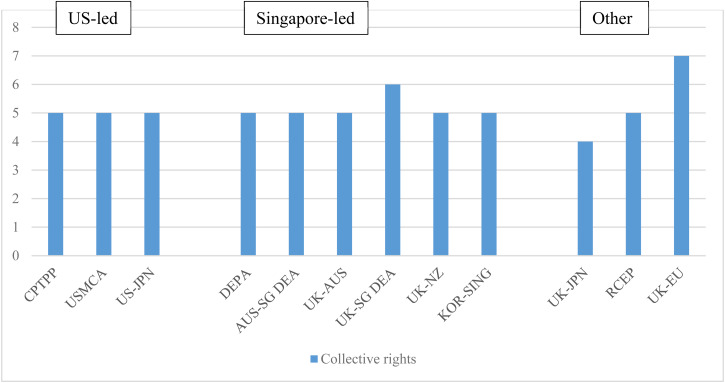
Extensiveness of collective rights commitments *Source:* Aggregation of legal coding of provisions by authors (see Table 1).

Although there are growing concerns about how to effectively protect labour rights in the context of a global digital economy where platform companies operate across borders, trade agreements are yet to address this issue.^183^ Among the agreements we reviewed, the Singapore–UK FTA was the only one to mention the issue, and it merely acknowledges the importance of adopting or maintaining policies that promote decent working conditions for digital economy workers, although the parties stop short of making any substantial commitments (Figure 6).^184^

### 4.6 Digital Inclusion and Stakeholder Consultation

The Singapore wave of digital agreements introduced provisions on stakeholder engagement and digital inclusion which were not present in the US-led agreements. These provisions were introduced through DEPA and have been incorporated in most subsequent agreements, although the commitments remain general in nature and are often non-binding.^185^

**Figure 7.**
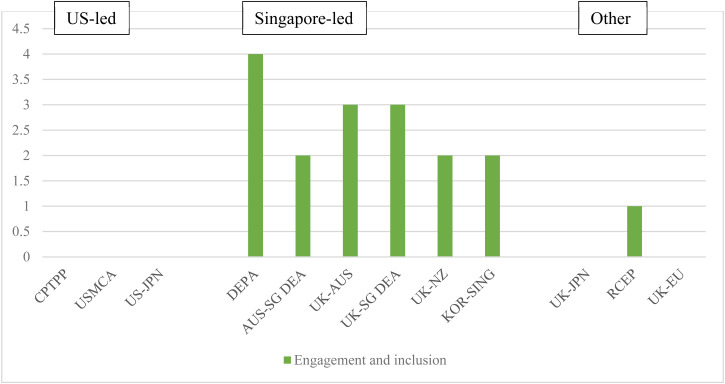
Extensiveness of commitments on digital inclusion and stakeholder consultation *Source:* Aggregation of legal coding of provisions by authors (see Table 1).

Provisions on stakeholder engagement acknowledge the importance of engaging a range of stakeholders in digital trade policymaking, and in some agreements, the parties commit to creating a forum or dialogue, where stakeholders can offer their input on issues related to digital trade.^186^ Commitments to digital inclusion aim to ensure that marginalized individuals and small and medium-sized enterprises can fully benefit from the digital economy.^187^ The provisions express the parties' recognition of the crucial role played by small and medium-sized enterprises in creating jobs and promoting economic growth, stating their intention to undertake activities to support them. In some instances, such as DEPA, Singapore–UK, and New Zealand–UK agreements, these provisions have been expanded to increase the participation of various groups in digital trade, including women, rural populations, and excluded socio-economic groups (Figure 7).^188^

## 5. Conclusion

The rapid advancement of data and digital technologies is causing a significant disruption in industries and supply chains worldwide. As a result, governments are updating trade rules to adapt to this transformation in international trade. We have examined the most recent wave of digital trade rulemaking, seeking to establish whether, and to what extent, it differs from the earlier US-led approach. Based on the examination and systematic coding of digital trade provisions in 12 agreements, we find substantial evidence of innovation, driven by Singapore. Drawing on the international relations literature on normative change, we identify Singapore as a normative entrepreneur, providing evidence that Singapore seeks to shape global norms and rules on digital trade, and forging digital trade agreements with like-minded countries is a core part of this strategy. Although normative change in digital trade is at an emergent stage, there is some evidence that the normative framing and rules that Singapore advocates are gaining traction, with some countries acceding to DEPA, and others, including the UK, emulating Singapore's approach in their own digital trade strategy.

We identify six agreements as part of a new ‘Singapore-led’ wave of digital trade agreements and provide a detailed analysis of how they compare with the earlier US-led approach. We find that the Singapore-led agreements largely follow the approach of the previous US-led agreements, replicating many of the specific and binding (and often controversial) rules found in US-led agreements, including on data flows and source code protection. However, they substantially extend the scope of digital rulemaking, covering 14 new issues, ranging from digital identities to governance of emerging technologies, and safety and security online. In doing so, they make greater efforts than the US-led agreements to promote regulatory alignment and interoperability. Despite the expansion of scope, provisions in important policy areas, such as competition in digital markets and labour rights in the gig economy, remain nascent, perhaps because these have not been a policy priority for Singapore or its allies.

Singapore's innovations have been welcomed by several other middle and small states, notably Australia, Chile, New Zealand, South Korea, and the UK, who have all entered into digital trade agreements with Singapore. Although there are signs that the norms and rules that Singapore has championed are gaining traction, it remains to be seen whether, and to what extent the innovations will be adopted by the world's major economies. In practice, it is uncertain whether these innovations will help preserve digital connectivity amidst heightened geopolitical tensions. Importantly, the Singapore-led approach does not (yet) offer a way to bridge some of the deep divergences and sources of tension in the global economy, including data governance and the regulation of frontier digital technologies.

## Supporting information

Jones et al. supplementary material Jones et al. supplementary material

## Footnotes

1 J.L. González and M.-A. Jouanjean (2017) ‘*Digital Trade: Developing a Framework for Analysis*’, OECD Trade Policy Papers 205, www.oecd-ilibrary.org/trade/digital-trade_524c8c83-en.

2 On the negotiation of e-commerce multilateral rules at the WTO, see S. Abendin and P. Duan (2021) ‘Global E-Commerce Talks at the WTO: Positions on Selected Issues of the US, European Union, China, and Japan’, World Trade Review 20, 707.

3 M.E. Janow and P.C. Mavroidis (2019) ‘Digital Trade, E-Commerce, the WTO and Regional Frameworks’, World Trade Review 18, S1; U. Dadush and E.D. Prost (2023) ‘Preferential Trade Agreements, Geopolitics, and the Fragmentation of World Trade’, World Trade Review 22, 278.

4 M. Burri, M.V. Callo-Müller and K. Kugler, ‘TAPED: Trade Agreement Provisions on Electronic Commerce and Data’, https://unilu.ch/taped.

5 H. Gao (2018) ‘Digital or Trade? The Contrasting Approaches of China and US to Digital Trade’, Journal of International Economic Law 21, 297.

6 M. Burri (2023) ‘A WTO Agreement on Electronic Commerce: An Inquiry into Its Legal Substance and Viability’, *Georgetown Journal of International Law*; M. Elsig and S. Klotz (2022) ‘Initiator Conditions and the Diffusion of Digital Trade-Related Provisions in PTAs’, International Interactions 48, 292.

7 The CPTPP text incorporates by reference the original TPP agreement although it also makes modifications to it.

8 M. Finnemore and K. Sikkink (1998) ‘International Norm Dynamics and Political Change’, International Organization 52, 887.

9 W.Y. Li (2023) ‘Regulatory Capture's Third Face of Power’, Socio-Economic Review 21, 1217.

10 S.J. Evenett and J. Fritz (2022) ‘Emergent Digital Fragmentation: The Perils of Unilateralism – A Joint Report of the Digital Policy Alert and Global Trade Alert’, Global Trade Alert; Dadush and Prost, supra n. 3; A. Bradford (2023) Digital Empires: The Global Battle to Regulate Technology. Oxford University Press.

11 Gao, supra n. 5; D. Erdos (2017) ‘Beyond “Having a Domestic”? Regulatory Interpretation of European Data Protection Law and Individual Publication’, *Computer Law & Security Review* 33, 275; J.P. Meltzer (2019) ‘Governing Digital Trade’, *World Trade Review* 18, S23.

12 G.H. Gao (2018) ‘Regulation of Digital Trade in US Free Trade Agreements: From Trade Regulation to Digital Regulation’, Legal Issues of Economic Integration 45, 47; R. Wolfe (2019) ‘Learning about Digital Trade: Privacy and E-Commerce in CETA and TPP’, *World Trade Review* 18, S63; S. Yakovleva and K. Irion (2020) ‘Pitching Trade Against Privacy: Reconciling EU Governance of Personal Data Flows With External Trade’, *International Data Privacy Law*, 10.; A. Chander and P. Schwartz (2023) ‘Privacy and/or Trade’, *The University of Chicago Law Review* 90, 49.

13 Gao, supra n. 5.

14 H. Gao (2018) ‘Regulation of Digital Trade in US Free Trade Agreements: From Trade Regulation to Digital Regulation’, *Legal Issues of Economic Integration* 45(1), at 47; S. Aaronson (2015) ‘What Does TPP Mean for the Open Internet?’, Institute for International Economic Policy, George Washington University; N. Mishra (2017) ‘The Role of the Trans-Pacific Partnership Agreement In the Internet Ecosystem: Uneasy Liaison or Synergistic Alliance?’, *Journal of International Economic Law* 20(1), 31–60, https://doi.org/10.1093/jiel/jgw080; M. Elsig and S. Klotz (2022) ‘Initiator Conditions and the Diffusion of Digital Trade-Related Provisions in PTAs’, *International Interactions* 48(2), 292–308, https://doi.org/10.1080/03050629.2022.2004137.

15 S. Aaronson and P. Leblond (2018) ‘Another Digital Divide: The Rise of Data Realms and Its Implications for the WTO’, *Journal of International Economic Law*, at 1; S. Bernstein and E. Hannah (2008) ‘Non-State Global Standard Setting and the WTO: Legitimacy and the Need for Regulatory Space’, Journal of International Economic Law 11(3), at 575.

16 S. Wunsch-Vincent and A. Hold (2012) ‘Towards Coherent Rules for Digital Trade: Building on Efforts in Multilateral Versus Preferential Trade Negotiations’, in M. Burri and T. Cottier (eds.), *Trade Governance in the Digital Age: World Trade Forum*. Cambridge University Press, 179–221.

17 M. Burri, M.V. Callo-Müller and K. Kugler (2022) ‘TAPED: Trade Agreement Provisions on Electronic Commerce and Data’, https://unilu.ch/taped (visited 12 December 2022).

18 M. Burri and R. Polanco (2020) ‘Digital Trade Provisions in Preferential Trade Agreements: Introducing a New Dataset’, *Journal of International Economic Law* 23(1), at 187; see Burri et al., supra n. 14.

19 M. Burri (2022) ‘Trade Law 4.0: Are We There Yet?’, *Journal of International Economic Law*, https://academic.oup.com/jiel/advance-article/doi/10.1093/jiel/jgac053/6965847 (accessed 21 February 2023); Burri, supra n. 6; M. Soprana (2021) ‘The Digital Economy Partnership Agreement (DEPA): Assessing the Significance of the New Trade Agreement on the Block’, *Trade Law and Development* 13, 143; S. Honey (2021) ‘Asia-Pacific Digital Trade Policy Innovation’, in I. Borchert and A. Winters (eds.), *Addressing Impediments to Digital Trade.* CEPR Press. https://cepr.org/publications/books-and-reports/addressing-impediments-digital-trade.

20 ‘Comprehensive and Progressive Agreement for Trans-Pacific Partnership (CPTPP)’ (2018).

21 ‘Agreement between the US of America, the United Mexican States, and Canada’ (2020).

22 ‘Trade Agreement between the US of America and Japan’ (2019).

23 ‘Digital Economy Partnership Agreement’ (DEPA) (2020).

24 ‘The Singapore–Australia Digital Economy Agreement’ (2020).

25 ‘Agreement between the United Kingdom of Great Britain and Northern Ireland and Japan for a Comprehensive Economic Partnership’ (2020).

26 ‘The Regional Comprehensive Economic Partnership (RCEP)’ (2020).

27 ‘Trade and Cooperation Agreement between the United Kingdom of Great Britain and Northern Ireland, of the One Part, and the European Union and the European Atomic Energy Community, of the Other Part’ (2021).

28 ‘Free Trade Agreement between the United Kingdom of Great Britain and Northern Ireland and Australia’ (2021).

29 ‘Free Trade Agreement between the United Kingdom of Great Britain and Northern Ireland and the Republic of Singapore’ (2022).

30 ‘Free Trade Agreement between the United Kingdom of Great Britain and Northern Ireland and New Zealand’ (2022).

31 ‘The Digital Partnership Agreement between the Government of the Republic of Korea and the Government of the Republic of Singapore’ (2023).

32 While we have captured most digital trade issues, the list of issues we analyse is not exhaustive. For instance, we have not analysed commitments on express shipments or submarine cables.

33 For instance, on liability of internet service providers for intellectual property violations, we examined Article 10.47 of the EU–Singapore trade deal, which has been incorporated by the Singapore–UK agreement.

34 K.W. Abbott and D. Snidal (2000) ‘Hard and Soft Law in International Governance’, International Organization 54, 421.

35 G.C. Shaffer and M.A. Pollack (2009) ‘Hard vs. Soft Law: Alternatives, Complements, and Antagonists in International Governance’, Minnesota Law Review 94, 706.

36 EU–UK TCA 2021, art 198.

37 We only assign stars where we coded the substantive provisions as binding (i.e., ‘2’, ‘3’, or ‘4’) as it is only in these situations that an exception alters the substantive obligation in a meaningful way.

38 For a helpful guide to different types of exceptions in international law see L. Bartels and F. Paddeu (2020) (eds.) Exceptions in International Law, 1st edn Oxford: Oxford University Press.

39 Appellate Body Report, *US – Standards for Reformulated and Conventional Gasoline*, WT/DS2/10/Add.7, 26 August 1997; Appellate Body Report, *US – Import Prohibition of Certain Shrimp and Shrimp Products*, WT/DS58/23, 26 November 2001.

40 RCEP, 2020, art 12.14.3.

41 Note that the figures only depict the aggregation of the numerical values assigned in the legal coding of the treaty commitments and do not factor in their associated public policy flexibilities. However, these are discussed extensively in the main text.

42 Virtual interview with two Singaporean trade negotiators, 7 December 2023.

43 I. Borchert and M. Morita-Jaeger (2021) ‘Taking Stock of the UK–EU Trade and Cooperation Agreement: Trade in Services and Digital Trade’, UKTPO Briefing Paper 53.

44 Burri, ‘Trade Law 4.0’, supra n. 19.

45 ‘Why Silicon Valley Wins Big from the USMCA’, *Financial Times*,12 December 2018; S. Azmeh, C. Foster, and J. Echavarri (2020) ‘The International Trade Regime and the Quest for Digital Free Trade’, *International Studies Review* 22, 671, 671–692; Li, supra n. 9).

46 Gao, supra n. 12.

47 Interview with Singaporean trade negotiator (in person, Geneva), 7 December 2023.

48 Bradford, zupra n. 10. M. Burri (2017) ‘The Governance of Data and Data Flows in Trade Agreements: The Pitfalls of Legal Adaptation’, *UC Davis Law Review* 51; G.W. Voss (2022) ‘Cross-Border Data Flows, the GDPR, and Data Governance’, *International Organisations Research Journal* 17, 56; Aaronson and Leblond, supra n. 15; U. Ahmed (2019) ‘The Importance of Cross-Border Regulatory Cooperation in an Era of Digital Trade’, *World Trade Review* 18, S99.

49 M. Ramesh (1995) ‘Economic Globalization and Policy Choices: Singapore’, Governance 8(2), at 243; M. Liang (2005) ‘Singapore's Trade Policies: Priorities and Options’, ASEAN Economic Bulletin 22(1), at 49.

50 For instance, Singapore ranks highest in the world on CISCO's digital readiness index: www.cisco.com/c/en/us/about/csr/research-resources/digital-readiness.html.

51 A. Hwee Min, ‘Singapore Seeks to Reinforce Role as Digital Hub, Signs Partnership with EU’, *CNA* (1 February 2023), www.channelnewsasia.com/singapore/singapore-seeks-reinforce-role-digital-hub-signs-partnership-eu-3246786 (accessed 15 June 2023).

52 Finnemore and Sikkink, supra n. 8.

53 Smart Nation and Digital Government Office (2018) ‘Smart Nation: The Way Forward’, www.smartnation.gov.sg/files/publications/smart-nation-strategy-nov2018.pdf (accessed 22 December 2023).

54 ‘Oral Reply to PQ on the State of Singapore Economy’, www.mti.gov.sg/Newsroom/Parliamentary-Replies/2019/07/Oral-reply-to-PQ-on-the-state-of-Singapore-economy.

55
Ibid.

56 Interview with two Singaporean trade negotiators (virtual), 7 December 2023.

57 ‘Annex B: Joint Ministerial Statement on the Substantial Conclusion of Digital Economy Partnership Agreement (‘DEPA’) Negotiations’ (2020), www.mti.gov.sg/Trade/Digital-Economy-Agreements/The-Digital-Economy-Partnership-Agreementon.

58 Interview with Singaporean trade negotiator (in person, Geneva), 7 December 2023.

59
Ibid.

60 See ‘WTO Electronic Commerce Negotiations: Updated Consolidated Negotiating Text – November 2023 (Restricted)’ (2023) INF/ECOM/62/Rev.5.

61 Finnemore and Sikkink, supra n. 8.

62 G. Floater (2022), ‘A Step Change in Modern Trade: The UK–Singapore Digital Deal’ (*LinkedIn*, 2 September 2022), www.linkedin.com/pulse/step-change-modern-trade-uk-singapore-digital-deal-graham-floater/ (accessed 22 December 2023).

63 Note that the figures in Section 4 only depict the aggregation of the numerical values assigned in the legal coding of the treaty commitments and do not factor in their associated public policy flexibilities. However, these are discussed extensively in the main text.

64 Note that DEPA does not include a commitment on electronic authentication and trust services.

65 E. Ganne and H. Nguyen, *Standards Toolkit for Cross-border Paperless Trade: Accelerating Trade Digitalisation Through the use of Standards*. WTO & ICC, www.wto.org/english/res_e/booksp_e/standtoolkit22_e.pdf.

66 Department for Business and Trade, ‘Singapore–UK Digital Economy Agreement: Final Agreement Explainer’, *GOV.UK*, www.gov.uk/government/publications/uk-singapore-digital-economy-agreement-explainer/uk-singapore-digital-economy-agreement-final-agreement-explainer (accessed 15 June 2023); ‘World's First Digital Trade Financing Pilot between MLETR-Harmonised Jurisdictions’, www.mas.gov.sg/news/media-releases/2021/worlds-first-digital-trade-financing-pilot-between-mletr-harmonised-jurisdictions (accessed 15 June 2023); F. Thompson, ‘Singapore Amends Law to Give eBLs and Other Electronic Trade Instruments Legal Footing’, *Global Trade Review (GTR)* (10 February 2021), www.gtreview.com/news/fintech/singapore-amends-law-to-give-ebls-and-other-electronic-trade-instruments-legal-footing/ (accessed 15 June 2023).

67 CPTPP 2018, art 14.9.

68 DEPA 2020, art 2.2.

69 KOR-SG 2023, art 14.12.3. This is consistent with previous Korea's FTAs, where paperless trade provisions were also prominent. See E.Y. Kim (2019) ‘E-Commerce in South Korean FTAs: Policy Priorities and Provisional Inconsistencies’, *World Trade Review* 18, S85.

70 NZ–UK 2022, art 15.10.

71 Singapore Customs (2018) ‘Going beyond the National Single Window’, *WCO News*, https://mag.wcoomd.org/magazine/wco-news-87/going-beyond-the-single-window/ (accessed 15 June 2023).

72 DEPA, art 7.1.

73 AUS–SG 2020, art 29.

74 See for instance Memorandum of Understanding on Digital Identities Cooperation’, *GOV.UK*, www.gov.uk/government/publications/memoranda-of-understanding-with-singapore-digital-trade-facilitation-digital-identity-and-cyber-security/memorandum-of-understanding-on-digital-identities-cooperation (accessed 22 March 2022).

75 DEPA, art 2.5.

76 BIS Innovation Hub (2021) ‘Nexus: A Blueprint for Instant Cross-Border Payments’.

77 DEPA, art 2.7.

78 AUS–SG, art 11.

79 World Economic Forum (2020) ‘*The Futute of the Last-Mile Ecosystem*’, www3.weforum.org/docs/WEF_Future_of_the_last_mile_ecosystem.pdf.

80 World Bank (2021) *World Development Report 2021: Data for Better Lives | Crossing borders*, https://wdr2021.worldbank.org/stories/crossing-borders/; OECD (2022) *The Value of Data in Digital-based Business Models: Measurement and Economic Policy Implications*, www.oecd.org/publications/the-value-of-data-in-digital-based-business-models-measurement-and-economic-policy-implications-d960a10c-en.htm.

81 See Aaronson and Leblond, supra n. 10, at 245. For a discussion about national security concerns and data privacy in light of trade provisions, see N. Zhang (2019) ‘Trade Commitments and Data Flows: The National Security Wildcard’, *World Trade Review* 18, S49.

82 ‘Mapping Commonalities in Regulatory Approaches to Cross-Border Data Transfers’, vol. 248 (2021) OECD Trade Policy Papers 248, www.oecd-ilibrary.org/trade/mapping-commonalities-in-regulatory-approaches-to-cross-border-data-transfers_ca9f974e-en (accessed 11 April 2023); Gao, supra n. 12); A. Chander and P. Schwartz (2023) ‘Privacy and/or Trade’, *The University of Chicago Law Review* 90, 49.

83 KOR–SG, art 14.16.

84 CPTPP 14.8.

85 Ibid. 14.8.

86 For example Ibid. 14.11.

87 C. Tikkinen-Piri, A. Rohunen and J. Markkula (2018) ‘EU General Data Protection Regulation: Changes and Implications for Personal Data Collecting Companies’, *Computer Law & Security Review* 34, 134.

88 DEPA, art 4.2.

89 OECD, ‘Open Government Data – OECD’, *OECD Home*, www.oecd.org/gov/digital-government/open-government-data.htm (accessed 23 May 2023).

90 JPN–US 2019, art 19.18.

91 DEPA, art 9.5.

92 NZ–UK, art 15.17.

93 B. Mercurio and R. Yu (2021) ‘Convergence, Complexity and Uncertainty: Artificial Intelligence and Intellectual Property Protection’, in S.–y. Peng, C.-F. Lin and T. Streinz (eds.), *Artificial Intelligence and International Economic Law*, 1st edn. Cambridge University Press, www.cambridge.org/core/product/identifier/9781108954006%23CN-bp-7/type/book_part, (accessed 20 February 2023); B. Bodó, K. Irion, H. Janssen, and A. Giannopoulou (2021) ‘Personal Data Ordering in Context: The Interaction of Meso-Level Data Governance Regimes with Macro Frameworks’, *Internet Policy Review*, 10, https://policyreview.info/articles/analysis/personal-data-ordering-context-interaction-meso-level-data-governance-regimes (accessed 23 May 2023).

94 J. Mazur and M. Słok-Wódkowska (2022) ‘Access to Information and Data in International Law’, *Nordic Journal of International Law*, 91, 310.

95 DEPA, art 9.4.

96 EU–UK TCA, art 201.

97 S. Yakovleva and K. Irion (2020) ‘Pitching Trade Against Privacy: Reconciling EU Governance of Personal Data Flows With External Trade’, *International Data Privacy Law* 10(3); A. Chander and U.P. Le (2014) ‘Breaking the Web: Data Localization vs. the Global Internet’, https://papers.ssrn.com/abstract=2407858; M. Burri (2017) ‘Current and Emerging Trends in Disruptive Technologies: Implications for the Present and Future of EU's Trade Policy’, Study for the European Parliament EP/EXPO/B/INTA/2017/06.

98 M. Burri (ed.) (2021) ‘Data Flows and Global Trade Law’, *Big Data and Global Trade Law*, 1st edn. Cambridge University Press, www.cambridge.org/core/product/identifier/9781108919234%23PTN-bp-1/type/book_part (accessed 11 April 2023); Wolfe, supra n. 12).

99 EU-UK TCA, art 202.

100 RCEP, art 12.15.

101 S. Wachter, B. Mittelstadt, and C. Russell (2021) ‘Why Fairness Cannot be Automated: Bridging the Gap Between EU Non-Discrimination Law and AI’, *Computer Law & Security Review* 41, at 105567; H. Margetts (2022) ‘Rethinking AI for Good Governance’, Daedalus 151(2), at 360.

102 E. Jones (2023) ‘Digital Disruption: Artificial Intelligence and International Trade Policy’, Oxford Review of Economic Policy 39(1), at 70.

103 CPTPP, art 14.17.

104 JPN–US, art 19.16.

105 K. Irion (2022) ‘Algorithms Off-limits?: If Digital Trade Law Restricts Access to Source Code of Software Then Accountability will Suffer’, FAccT’22: 2022 ACM Conference, at 1561, https://doi.org/10.1145/3531146.3533212.

106 See Jones, supra n. 84.

107 According to Mishra, exceptions can offer a degree of flexibility to facilitate the regulation of AI, although in reality it may prove difficult for the WTO and other international trade arbitration bodies to differentiate between reasonable and overly protectionist policies, due to the complex and uncertain technical and policy considerations involved in the matter. N. Mishra (2021) ‘International Trade Law Meets Data Ethics: A Brave New World’, *NYU Journal of International Law* 53, 305.

108 Irion Kristina, supra n. 12).

109 In the Matter of the Treaty of Waitangi Act 1975 and the Trans-Pacific Partnership Agreement Inquiry, 2020, affidavit of Elizabeth Jane Kelsey, Professor at the University of Auckland. A. Sykes (2020) ‘Closing Submissions,’ in the Matter of The Treaty of Waitangi and the Trans-Pacific Partnership Agreement Inquiry.

110 A.O. Sykes (2021) ‘The Law and Economics of “Forced” Technology Transfer and Its Implications for Trade and Investment Policy (and the U.S.–China Trade War)’, Journal of Legal Analysis 13(1), at 127.

111 AUS–SG, art 28.

112 JPN–UK, art 8.73.

113 EU–UK TCA, art 207.

114 AUS–UK FTA 2021, art 14.18.

115 AUS–SG, art 8.61K.

116 KOR–SG, art 14.19.

117 See Irion, supra n. 74.

118 Frank Pasquale (2017) ‘Secret Algorithms Threaten the Rule of Law’, MIT Technology Review (1 June 2017) www.technologyreview.com/2017/06/01/151447/secret-algorithms-threaten-the-rule-of-law/.

119 Our analysis has also covered provisions specifically related to access to source code for cryptography in information and communication services.

120 There are also implications of cryptography on national security and export controls. Cryptography has both civilian and military applications, making it a dual-use technology that is regulated as such. Both the US and the EU adhere to the same multilateral export control regime for cryptography products, but they differ in their approach to implementation. The US links dual-use with national security interests in the context of the war on terror, while the EU sees human security as a critical argument for promoting encryption technologies. See V. Vella (2017) ‘Is There a Common Understanding of Dual-Use?: The Case of Cryptography’, *Strategic Trade Review* 3, https://orbi.uliege.be/handle/2268/259287 (accessed 23 May 2023).

121 H. Abelson et al. (2015) ‘Keys Under Doormats: Mandating Insecurity by Requiring Government Access to All Data and Communications’, *Journal of Cyber Security*, at tyv009.

122 CPTPP, art 11.7.

123 L.A. Estoup (2019) ‘FinTech under the New US–Mexico–Canada (USMCA) Agreement’, *Practical Law* (blog), http://uk.practicallaw.thomsonreuters.com/w-020-8365.

124 CPTPP, art 11.7.

125 Meltzer, supra n. 11).

126 AUS–SG, art 30; SG–UK 2022, art 8.61-D; KOR–SG, art 14.31.

127 KOR–SG, art 14.31.

128 For a detailed discussion on AI and digital trade, see Jones, supra n. 73.

129 DEPA, art 8.2; AUS–SG, art 31.

130 AUS–SG, art 20.4. Note that this provision is found in the innovation chapter, which is not subject to dispute settlement.

131 Ibid. 8.61.

132 NZ–UK, art 15.19.

133 Ibid. 15.19; 15.19.4(b).

134 For a discussion of lawtech uses and its limitations, see J. Armour and M. Sako (2021) *Lawtech: Levelling the Playing Field in Legal Services?*, SSRN, http://dx.doi.org/10.2139/ssrn.3831481.

135 T. Butler and L. O'Brien (2019) ‘Understanding RegTech for Digital Regulatory Compliance’, in T. Lynn et al. (eds.), *Disrupting Finance*. Cham: Springer International Publishing at 85.

136 AUS–SG, art 32.

137 Ibid. 8.53.

138 Ibid., art 8.61-T.

139 The analysis of provisions related to digital platforms also encompassed provisions on internet access, as well as commitments pertaining to network neutrality and non-discriminatory access.

140 J.-U. Franck and M. Peitz (2023) ‘Market Power of Digital Platforms’, Oxford Review of Economic Policy 39(1) (2023), at 34.

141 A. Fletcher (2023), ‘International Pro-Competition Regulation of Digital Platforms: Healthy Experimentation or Dangerous Fragmentation?’, Oxford Review of Economic Policy 39(1), at 12. For a discussion of intertwined data protection and competition concerns that emerge in digital markets, see B. Kira, V. Sinha, and S. Srinivasan (2021) ‘Regulating Digital Ecosystems: Bridging the Gap Between Competition Policy and Data Protection’, Industrial and Corporate Change 30(4), at 1337.

142 J. Riordan (2020) ‘A Theoretical Taxonomy of Intermediary Liability’, in G. Frosio (ed.), Oxford Handbook of Online Intermediary Liability. Oxford University Press at 56.

143 Japan–US 2019, art 18. The TPP deal included rules on general intermediary liability in article 18.82, but this provision was fully suspended by the CPTPP.

144 USMCA 2022, art 19.17.

145 JPN–US, art. 18.

146 G. Frosio (2020) ‘Mapping Online Intermediary Liability’, in G. Frosio (ed.), Oxford Handbook of Online Intermediary Liability. Oxford University Press at 1.

147 Chander, supra n. 143). At the domestic level, the very scope and application of section 230 CDA immunity was recently discussed by the US Supreme Court, although the Court declined to decline to address the application of §230 in that case. See *Gonzalez v. Google LLC – Supreme Court of the US*, www.supremecourt.gov/opinions/22pdf/21-1333_6j7a.pdf (visited 14 June 2023).

148 P. Samuelson (2021) ‘Pushing Back on Stricter Copyright ISP Liability Rules’, *Michigan Technology Law Review* 27, 299.

149
Ibid.

150 A. Marsoof and I. Gupta (2019) ‘Shielding Internet Intermediaries from Copyright Liability—A Comparative Discourse on Safe Harbours in Singapore and India’, *The Journal of World Intellectual Property* 22, 234.

151 While general safe-harbour provisions shielding internet companies from liability have not been adopted beyond US-led deals, provisions that establish safe harbours for intellectual property violations are more commonplace and have been found in both US-led and Singapore-led agreements. Such provisions often feature in the intellectual property chapter of trade deals, and are generally binding and prescriptive, offering no space for parties to depart from the liability model they establish.

152 D.K. Citron and B. Wittes (2017) ‘The Internet Will Not Break: Denying Bad Samaritans § 230 Immunity’, *Fordham Law Review* 86, 401.

153 DEPA, art 5.2.

154 AUS–SG, art 18.1–18.4.

155 CPTPP, art 14.10.

156 USMCA, art 19.10.

157 On network neutrality commitments in the TPP, see Mishra, supra n. 14).

158 The US Federal Communications Commission (FCC) changed American ISPs rules in 2017, *de facto* repealing the network neutrality principle in the country. Aaronson and Leblond, supra n. 15). The US Federal Communications Commission (FCC) changed American ISPs rules in 2017, *de facto* repealing the network neutrality principle in the country. S.A. Aaronson and P. Leblond (2018) ‘Another Digital Divide: The Rise of Data Realms and Its Implications for the WTO’, *Journal of International Economic Law* 21, 245.

159 DEPA, art 6.4.

160 AUS–SG ch 14:20.

161 KOR–SING, art. 14.24.

162 JPN–UK CEPA, art 8.78.

163 EU–UK TCA, art 5.33.

164 R.S. Neeraj (2019), ‘Trade Rules for the Digital Economy: Charting New Waters at the WTO’, *World Trade Review* 18, S121; Fletcher, supra n. 141.

165 AUS–SG, art 16.

166 AUS–UK FTA, art 17.6.

167 SG–UK, art 8.61.

168 NZ–UK, art 18.5.

169 DEPA, art 8.4.

170 See Mishra, supra n. 7, 45; S.A. Aaronson (2021) ‘Can Trade Agreements Help Solve the Wicked Problem of Disinformation?’, SSRN, https://papers.ssrn.com/abstract=3820213.

171 CPTPP, art 14.7.

172 DEPA, art 6.3.

173 NZ–UK, art 203.

174 Ibid. 20.5.

175 USMCA, art 19.13.

176 Mishra, supra n. 14.

177 AUS–SG, art 14.17.

178 EU–UK TCA, art 209.

179 CPTPP, art 14.16.

180 USMCA, art 19.

181 SG–UK, art 8.61-L.

182 Mishra, supra n. 14, 46.

183 ILO WorkQuality Department and DDG/P (2019) ‘Policy Responses to New Forms of Work: International Governance of Digital Labour Platforms’, www.ilo.org/global/about-the-ilo/how-the-ilo-works/multilateral-system/g20/reports/WCMS_713362/lang--en/index.htm (accessed 16 June 2023).

184 SG–UK, art 8.61P.

185 DEPA, art 10.4.

186 Ibid.; AUS–SG, art 35; RCEP, art 12.16; AUS–UK FTA, art 20.5; AUS–SG, art 20.5; SG–UK, art 8.61trV; KOR–SG, art 14.34. Note that the provision in the AUS–UK FTA is found in the innovation chapter, which is not subject to dispute settlement.

187 DEPA, art 10.4; AUS–SG, art 35; RCEP, art 12.16; AUS–UK FTA, art 20.5; AUS–SG, art 20.5; SG–UK, art 8.61-V; KOR–SG, art 14.34.

188 DEPA, art 10.4; SG–UK, art 8.61-V.

## Supplementary Materials

To view supplementary material for this article, please visit https://doi.org/10.1017/S1474745624000089.

