# Supplementary material for: Norm Entrepreneurship in Digital Trade: The Singapore-led Wave of Digital Trade Agreements
Source: World Trade Rev. 2024 Mar 20;23(2):208–41. doi: 10.1017/S1474745624000089 (PMC11451010; doi:10.1017/S1474745624000089)
Supplement: Jones et al. supplementary material [file S1474745624000089sup001.docx]

**ANNEX**

**Codebook – digital trade provisions in US-led and Singapore-led agreements**

**Dataset and indicators**

This document is the authoritative codebook for the database covering digital trade provisions in 12 agreements which have been concluded between March 2018 and January 2023 and have extensive digital commitments. In chronological order, the agreements included in the dataset are:

- Comprehensive and Progressive Agreement for Trans-Pacific Partnership (CPTPP, March 2018)
- United States-Mexico-Canada Agreement (USMCA, November 2018)
- Japan-U.S. Digital Trade Agreement (JPN-US, October 2019)
- Digital Economic Partnership Agreement (DEPA, June 2020)
- Australia-Singapore Digital Economy Agreement (AUS-SG, August 2020)
- Japan-UK Free Trade Agreement (JPN-UK, October 2020)
- Regional Comprehensive Economic Partnership (RCEP, November 2020)
- EU-UK Trade and Cooperation Agreement (EU-UK, December 2020)
- Australia-UK Free Trade Agreement (AUS-UK, December 2021)
- Singapore-UK Digital Economy Agreement (SG-UK, February 2022)
- New Zealand-UK Free Trade Agreement (NZ-UK, February 2022)
- Korea-Singapore Digital Economy Agreement (KOR-SG, January 2023)

While other trade agreements have been concluded during this period, we excluded those without substantial digital chapters from our analysis, as our aim is to understand the scope and nature of the frontier of rulemaking in digital trade, rather than provide an exhaustive analysis of all digital provisions in recent trade agreements.

The dataset contains 34 indicators organised into six groups:

1. Trade facilitation
   1. Upholding the moratorium on customs duties on e-transmissions
   2. Domestic electronic transactions framework
   3. Conclusion of contracts by electronic means
   4. E-authentication and trust services
   5. Facilitating paperless trade
   6. Cooperation and interoperability on digital identities
   7. Implementation of e-invoicing systems
   8. Regulating cross-border e-payments
   9. Regulating cross-border logistics
2. Cross-border data flows
   1. Free flow of data (non-financial)
   2. Data localisation (non-financial)
   3. Protection of personal information
   4. Free flow of financial data
   5. Localisation of financial data
   6. Access to government data
   7. Supporting data innovation
3. Innovation and regulation of new technologies
   1. Mandatory disclosure of source code
   2. Regulation of ICT products that use cryptography
   3. Adoption of standards & conformity assessment
   4. Governance of AI and emerging technologies
   5. Supply of new financial services
   6. Collaboration on regtech
   7. Lawtech cooperation
4. Regulation of digital platforms
   1. Limiting ISP liability for online harms (non-IP)
   2. Limiting ISP liability for IP infringement
   3. Safety and security online
   4. Principles on access to and use of the internet
   5. Cooperation on competition policy in digital markets
5. Collective rights
   1. Protection of online consumers
   2. Regulation of spam
   3. Cooperation on cybersecurity matters
   4. Labour protections
6. Stakeholder engagement, SMEs and digital inclusion
   1. Promoting stakeholder engagement
   2. Facilitating Digital inclusion

The database includes provisions from digital trade chapters as well as provisions in other chapters that specifically relate to digital trade (for instance, commitments on cross-border flows of financial data are often found in the financial services chapter, while commitments on the liability of internet service providers are typically found in intellectual property chapters). Where a digital agreement is being negotiated to update and amend an underlying FTA, we examine the original treaty to ensure we included any provisions that are incorporated (or excluded) by reference. For instance, on liability of internet service providers for intellectual property violations, we examined Article 10.47 of the EU–Singapore trade deal, which has been incorporated by the Singapore–UK agreement by reference.

**Coding**

For each indicator, in each trade agreement examined, our coding captures two dimensions: (i) prescriptiveness of the legal commitment, and (ii) public policy flexibility embedded in the provision, as detailed below.

*Prescriptiveness of the legal commitment*

The prescriptiveness dimension assesses how substantive the obligation established in the provision is. This is recorded on an ordinal scale that represents the level of obligation agreed by the parties. Importantly, the coding does not reflect any normative assessment of the content of the obligation, but merely how strict and specific the obligation placed on the parties is. In other words, the coding does not represent a judgement as to the economic or societal value of the commitment (e.g. whether it is more or less liberalising, or more or less protective of consumers), but rather assess its level of prescriptiveness.

| **Coding** | **Description** | **Example and rational** |
| --- | --- | --- |
| 0 – Non-existent | The agreement does not include a provision on that issue, which has been found in other agreements. | NZ-UK does not include a provision on source code disclosure. Regardless of the merit of the exclusion of the provision from the agreement, it is coded as 0 as it is not present in the examined text |
| 1 – Not Binding | The provision is couched in soft language that does not amount to a binding obligation, including best endeavours provisions and other forms of non-binding commitments. | DEPA provision on cross-border logistics is drafted in purely hortatory language, mentioning that Parties ‘recognise the importance of’ and ‘shall endeavour to’, reflecting the non-binding nature of the commitment. |
| 2 – Binding and Vague | The provision establishes some sort of binding obligations, but these are weak and there are also important gaps regarding the breadth of the provision, as well as the level of nuance with which the issue was addressed. Provisions where parties agree to cooperate or to exchange information fall within this description. | NZ-UK provision on customs duties mentions that ‘Parties shall cooperate in relevant international fora to promote the adoption of commitments by non-parties not to impose customs duties on electronic transmissions.’ This would be understood as a binding obligation given the language (‘shall’), even though the core of the obligation might be soft in nature (i.e., cooperation). |
| 3 – Binding and Specific | The provision establishes binding and substantive obligations, describing actions to be taken (or not taken) in clear and precise language, with some level of specificity. | JPN-UK provision on new financial services establishes that parties “shall permit financial service suppliers of the other Party to offer in its territory any new financial service that the Party would permit its own financial service suppliers, in like situations, to supply without adopting or modifying a law.” Because the obligation is binding and provides details about how it should be fulfilled, it is substantive and specific. |
| 4 – Binding and Extensive | The provision establishes binding and very substantive obligations, describing actions to be taken (or not taken) in clear and precise language, with high level of specificity and details. | AUS-SG commitment on internet service liability for intellectual property violation commit parties to adopt a very specific regulatory model. Because the provision is binding and provides a great level of detail about the rules that should be adopted by the parties, including rules on available remedies and defences, it is considered comprehensive. |

*Public policy flexibility*

This dimension assesses the exceptions to the obligations established by the provision, capturing the level of flexibility granted in the provision that would allow parties to adopt measures to achieve public policy objectives, that is, how much parties would be allowed to depart from the obligations. When assessing the level of public policy flexibility, the coding reflects such as which authorities have the power to use the carve-out, what aspects of the provision are subject to a carve-out, and how extensive can the carve-out be.

Importantly, the coding only reflects the level of public policy flexibility that is provided by the specific provision under examination and does not reflect the flexibility provided by any agreement-wide or chapter-wide general exceptions. In some cases, states introduce additional flexibility by excluding the digital provisions from dispute settlement, notably in the case of the e-commerce chapter in RCEP and the innovation chapter in the AUS-UK agreement. When the provision is excluded from dispute settlement, we present the coding between rounded bracket (*coding* *value*).

| **Coding** | **Description** | **Example and rational** |
| --- | --- | --- |
| No * – Non-existent | The provision does not include any mentions to public policy exceptions or carve-outs whatsoever. The agreement might include agreement-wide or chapter-wide exceptions, but there are no specific exceptions or carve-out in relation to the specific provision coded. | The USMCA provision on general liability of internet service providers establishes detailed binding obligations to the parties, but does not include any exceptions or carve-outs that would allow them to depart from the prescriptive regime defined in the agreement. |
| *  –  Limited | The exceptions and carve-outs are limited in scope, or the qualifying criteria are relatively stringent, giving parties limited flexibility to regulate on public policy grounds in narrow circumstances. | The JPN-UK provision banning mandatory disclosure of source code includes a carve-out establishing that the article “does not preclude a regulatory body or judicial authority” from requiring the source code “for a specific investigation, inspection, examination, enforcement action, or judicial proceeding”. Because there are few authorities and situations covered by the exception, this is considered a limited carve-out. |
| ** – Wide | The exceptions or carve-outs are expansively drafted and give Parties a wide array of discretion to regulate on public policy grounds. This includes language allowing Parties to breach the rule for any ‘legitimate public policy’ reason without further qualification, or where the rule is subject to a broad qualification such as ‘except as otherwise provided for in its laws and regulations’. | UK-SG provision on paperless trading has a carve-out in cases where “there is a domestic or international legal requirement to the contrary” or when performing the obligation “would reduce the effectiveness or the trade administration process.” Because the exceptions are open-ended and welcomes political considerations, the carve-out is considered wide. |
| *** – Entirely Self Judging Carve-out | The exceptions or carve-outs are expansively drafted and entirely self-judging, giving Parties discretion to determine whether or not the provision applies. This means that the other parties to the agreement may have little or no recourse to challenge or dispute the invocation of the carve-out. | RCEP provision on data flows commit parties allow cross-border transfer of information, but it includes not only a general public policy carve-out but also an exception allowing parties to adopt “any measure that it considers necessary for the protection of its essential security interests”, further stating that “such measures shall not be disputed by other Parties.” Because the party can decide for itself whether a measure is essential to protect security interests, and this decision cannot be contested, this is an entirely self-judging exception. |
